# MyoD is a 3D genome structure organizer for muscle cell identity

**DOI:** 10.1038/s41467-021-27865-6

**Published:** 2022-01-11

**Authors:** Ruiting Wang, Fengling Chen, Qian Chen, Xin Wan, Minglei Shi, Antony K. Chen, Zhao Ma, Guohong Li, Min Wang, Yachen Ying, Qinyao Liu, Hu Li, Xu Zhang, Jinbiao Ma, Jiayun Zhong, Meihong Chen, Michael Q. Zhang, Yong Zhang, Yang Chen, Dahai Zhu

**Affiliations:** 1 The State Key Laboratory of Medical Molecular Biology, Institute of Basic Medical Sciences, Chinese Academy of Medical Sciences and School of Basic Medicine, Peking Union Medical College, 5 Dong Dan San Tiao, 100005 Beijing, China; 2 MOE Key Laboratory of Bioinformatics, Center for Synthetic and Systems Biology, Bioinformatics Division, BNRist, Department of Automation, Tsinghua University, 100084 Beijing, China; 3 MOE Key Laboratory of Bioinformatics, Center for Synthetic and Systems Biology, School of Medicine, Tsinghua University, 100084 Beijing, China; 4 Department of Biomedical Engineering, College of Future Technology, Peking University, 100871 Beijing, China; 5 Department of Biomedical Engineering, College of Engineering, Peking University, 100871 Beijing, China; 6 National Laboratory of Biomacromolecules, CAS Center for Excellent in Biomacromolecules, Institute of Biophysics, Chinese Academy of Sciences, 100101 Beijing, China; 7 University of Chinese Academy of Science, 100049 Beijing, China; 8 Bioland Laboratory (Guangzhou Regenerative Medicine and Health Guangdong Laboratory), 510320 Guangzhou, China; 9 Beijing institute of collaborative innovation, 100094 Beijing, China; 10 State Key Laboratory of Genetic Engineering, Department of Biochemistry, School of Life Sciences, Fudan University, 200438 Shanghai, China; 11 Department of Biological Sciences, Center for Systems Biology, The University of Texas, Dallas 800 West Campbell Road, RL11, Richardson, TX 75080-3021 USA

**Keywords:** Differentiation, Chromatin structure, Muscle stem cells

## Abstract

The genome exists as an organized, three-dimensional (3D) dynamic architecture, and each cell type has a unique 3D genome organization that determines its cell identity. An unresolved question is how cell type-specific 3D genome structures are established during development. Here, we analyzed 3D genome structures in muscle cells from mice lacking the muscle lineage transcription factor (TF), MyoD, versus wild-type mice. We show that MyoD functions as a “genome organizer” that specifies 3D genome architecture unique to muscle cell development, and that H3K27ac is insufficient for the establishment of MyoD-induced chromatin loops in muscle cells. Moreover, we present evidence that other cell lineage-specific TFs might also exert functional roles in orchestrating lineage-specific 3D genome organization during development.

## Abstract (web summary)

Pioneer transcription factors (TFs) have been proposed to act as protein anchors to orchestrate cell type-specific 3D genome architecture. MyoD is a pioneer TF for myogenic lineage specification. Here the authors provide further support for the role of MyoD in 3D genome architecture in muscle stem cells by comparing MyoD knockout and wild-type mice.

## Introduction

Recent Hi-C analysis of comprehensive interaction maps over large regions or whole genomes has indicated that the genome is hierarchically organized into chromosome territories, A/B compartments, topologically associated domains (TADs), and chromatin loops^1–3^. Many studies have shown that the 3D structure of the genome differs widely among different cell types^4–6^. However, the molecular mechanisms underlying the establishment of cell-type-specific 3D genome organization are largely unknown. It has been proposed that “master transcription factors” (TFs) act as anchor proteins to orchestrate cell-type-specific 3D genome architecture^7,8^. For example, studies have shown that KLF4 is involved in the organization and regulation of pluripotency-associated three-dimensional enhancer networks^9^, LHX2- and LDB1-mediated *trans*-interactions regulate olfactory receptor choice^10^, and Pax3 cooperates with Ldb1 to direct local chromosome architecture during myogenic-lineage specification^11^.

MyoD (myogenic differentiation 1) and Myf5 are members of the myogenic regulatory factor (MRF) family and are expressed in somites^12–15^. These proteins function as a basic helix–loop–helix transcription factors and are required for myogenic determination during early embryogenesis. This was initially evidenced by the complete blockade of skeletal muscle formation in *MyoD* and *Myf5* double-mutant mice^16^. Later studies demonstrated that the phenotype observed in Myod-Myf5 double-knockout (KO) mice was caused by impaired expression of Mrf4^16,17^. MyoD and Myf5 synergistically regulate the expression levels of myogenic genes genome-wide by binding their consensus E-box sequences^18^. Previous work and our present data indicate that *Myf5* expression is significantly upregulated in *MyoD*-null cells^19^. The single inactivation of either MyoD or Myf5 in mice results in apparently normal muscle development but delayed myogenic differentiation^19–22^ and impaired muscle regeneration^20^. Together, these observations indicate that MyoD and Myf5 can functionally compensate for one another during myogenic development^17,23^.

In this study, we explored the cell-lineage-specific master transcription factor-mediated specification of developmental-context-specific 3D genome organization, with a focus on MyoD. We chose MyoD for the following reasons: First, MyoD has been well-established as a master TF in myogenic cell-lineage specification during development^16,24–26^ and *trans*-differentiation^27^. It is known to regulate the expression of myogenesis-specific genes through binding to consensus E-box (CANNTG)-containing *cis*-regulatory elements^28,29^, which number more than 14 million across the genome^28^. Second, over 40,000 MyoD-binding peaks have been identified by independent ChIP-seq studies^29–31^ in muscle cells; of them, only 15% are located in promoter regions (Supplementary Fig. 1a). Finally, in addition to its functions in binding and *trans*-activating genes during differentiation, MyoD also constitutively binds at tens of thousands of additional sites throughout the genome in proliferating muscle stem cells^29,32^ (Supplementary Fig. 1a). Together, these data emphasize the attractively large empirical scope represented by genome-wide functional analysis of the potential roles of MyoD in muscle-cell-lineage-specific 3D genome organization. In this report, we provide computational and experimental evidence that uncovers a previously unappreciated role of MyoD as a genome organizer that contributes to establishing the unique 3D genome architecture in muscle cells, beyond its well-known functions as a TF in activating myogenic gene expression during development.

## Results

### MyoD regulates A/B compartment switching and the formation of contact domain boundaries (CDBs) in muscle cells

**Fig. 1:**
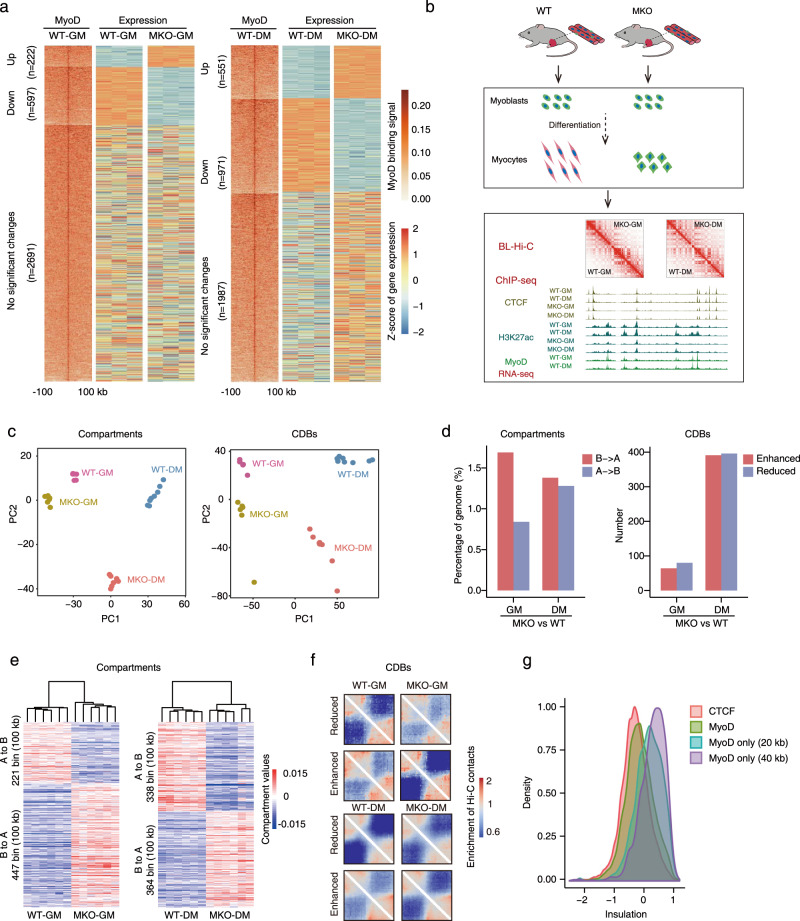
MyoD regulates A/B compartment switching and contact domain boundary (CDB) formation in muscle cells. **a** Association of MyoD-binding peaks at promoter regions and related gene expression. Heatmaps show the MyoD-binding peaks at promoters (centralized at the TSS within 3 kb distance) and related gene expression trends in WT and MKO myoblasts (left panel). Corresponding data for WT and MKO myocytes is shown in the right panel. **b** Strategy for genetic investigation of the chromatin architectural roles of MyoD. **c** Principal Component Analysis (PCA) of the values of compartments or the relative insulation scores of contact domain boundaries (CDBs) among the four indicated sample types. Each dot represents an individual biological replicate. **d** Bar plots showing the percentage of A/B compartment shifts between WT and MKO myoblasts and myocytes (left panel). Bar plots showing the number of relative insulation-enhanced or -reduced CDBs between WT and MKO in proliferating and differentiating muscle stem cells (right panel). **e** Heatmaps of the A/B compartment-shifted regions between WT and MKO myoblasts and myocytes. **f** Hi-C aggregation plots centered at differential CDBs between WT and MKO myoblasts and myocytes. **g** Probability density of insulation score at genetic regions bound by CTCF or MyoD. Four classes were assessed: CTCF-bound; MyoD-bound; MyoD-bound; and CTCF-unbound within a 20 kb distance; and MyoD-bound and CTCF-unbound within a 40 kb distance.

In light of the aforementioned MyoD data, we first performed RNA-seq analysis of muscle cells that were isolated from hind-limb skeletal muscles of wild-type (WT) and *MyoD*-null (MKO) mice and cultured in growth medium (GM, Pax7^+^, and MyoD^+^) or differentiation medium (DM, MyoG^+^) (Supplementary Fig. 1b). We then combined our results with existing MyoD ChIP-seq data to examine potential functional link(s) between MyoD occupancy and transcriptional activation. We extracted MyoD-binding peaks localized at promoter regions and identified their cognate genes within ±3 kb (Fig. 1a). Intriguingly, we found that knockout of MyoD did not affect the expression of a majority of these genes (2691/3510 (76.7%) in primary proliferating myoblasts, 1987/3509 (56.6%) in differentiating myocytes) (Fig. 1a and Supplementary Data 1), suggesting that the presence of MyoD at these genes (even in their promoter regions) frequently does not directly impact their transcriptional activation. This result further supports the hypothesis that, beyond its canonical function as an activator of myogenic gene expression during development, MyoD may exert broad functional impacts, potentially on higher-order genome structures.

To directly investigate the potential genome architectural roles of MyoD in muscle cells, we examined muscle samples from four groups (WT-GM, WT-DM, MKO-GM, and MKO-DM) using bridge-linker Hi-C (BL-Hi-C)^33^, which is a modified in situ Hi-C with improved sensitivity and specificity for structural and regulatory chromatin loop detection. In the BL-Hi-C experiments, 28 libraries were sequenced to a total depth of over 12 billion reads. The high-quality deep-sequencing data were validated with a high *cis*-interaction rate (Supplementary Fig. 1c) and reached 5 kb resolution (Supplementary Fig. 1d), which ensured the rigor of our subsequent computational analyses for each hierarchical level of chromatin structure in muscle cells. As an integrative analysis of the function of MyoD in orchestrating 3D genome organization, we also performed ChIP-seq for CTCF (CCCTC-binding factor) and H3K27ac (histone H3 lysine 27 acetylation) (Fig. 1b).

We then examined the impacts of MyoD depletion on chromatin A/B compartments in muscle cells by performing the exploratory principal component analysis (PCA) based on the compartment values for the four muscle sample types examined. We observed clear distinctions between the WT and MKO genotypes for both proliferating and differentiating muscle cells (Fig. 1c). Moreover, we detected a clear A/B compartment shift in proliferating MKO cells vs. WT cells (1.69% B→A, 0.84% A→B) (Fig. 1d left and 1e left). For differentiating cells, there was a clear compartment switch for MKO cells compared to differentiating WT cells (1.38% B→A, 1.28% A→B) (Fig. 1d left, and 1e right).

We also analyzed the dynamics of chromatin contact domain boundaries (CDBs)^34^ in muscle cells in response to MyoD knockout (Fig. 1c right). A total of 931 CDBs exhibited differential extents of insulation in MKO cells compared to WT cells in the proliferating and differentiating states (Fig. 1d right and f), implying that MyoD may somehow facilitate CDB insulation. Pursuing this, we compared insulation scores for CDBs bound by MyoD or CTCF. In general, the insulation scores of MyoD-bound CDBs were similar to those of CTCF-bound CDBs (Fig. 1g). However, the CDBs bound solely by MyoD exhibited significantly weaker insulation compared to the CTCF-bound CDBs. This indicates that MyoD may work in concert with CTCF to regulate the insulation of CDBs in muscle cells.

### MyoD is an anchor protein for chromatin loop formation in muscle cells

**Fig. 2:**
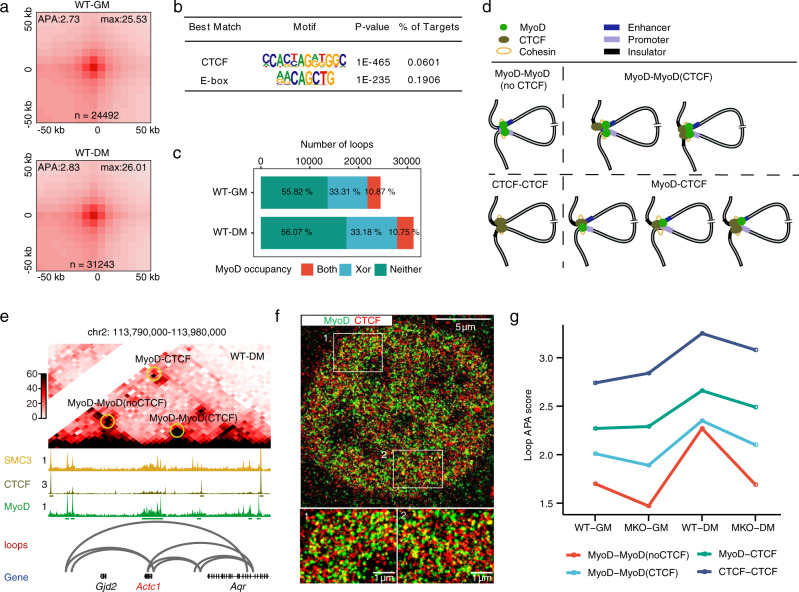
MyoD is an anchor protein for chromatin loop formation in muscle cells. **a** Aggregate Peak Analysis (APA) plots showing the aggregated Hi-C contacts around chromatin loops identified in WT-GM and WT-DM cells. *n* represents the total number of chromatin loops called by HiCCUPS (see Methods). The APA score at the upper left corner of each aggregation plot was calculated as the ratio of Hi-C contacts at the central pixel to the mean Hi-C contacts of the lower-left pixels. The Hi-C contacts at the central pixel are shown at upper right corner of each aggregation plot. **b** Enriched motifs in accessible regions at the anchors of the chromatin loops identified in panel **a**. *p*-values of motif enrichment were determined using cumulative binomial distributions, as applied by Homer. **c** Percentage of chromatin loops with anchors bound by MyoD in WT-GM and WT-DM cells. MyoD is indicated as being bound at both anchors (Both, red), one of the two anchors (Xor, blue), or neither of the two anchors (Neither, green). Pseudo-peaks of MyoD binding derived from combined ChIP-seq data of public databases and our study were used for this analysis (Methods). **d** Classification of the four types of chromatin loops identified in the study. MyoD-MyoD(noCTCF) loops had both anchors bound only by MyoD. MyoD-MyoD(CTCF) loops had both anchors bound by MyoD and either anchor concomitantly bound by CTCF. MyoD-CTCF loops had only one anchor bound by MyoD and either anchor bound with CTCF. CTCF-CTCF loops had both anchors bound only by CTCF. **e** Representative examples of the MyoD-bound chromatin loops. ChIP-seq data of SMC3 in C2C12 cells were obtained from the public database (GSE113248). **f** dSTORM image showing colocalization of MyoD (green) and CTCF (red) from immunofluorescent staining of C2C12 cells grown in DM for 24 h and stained with antibodies against MyoD and CTCF. The image is representative of three independent experiments. **g** APA score dynamics for the four types of chromatin loops among WT and MKO cells grown in GM and DM.

The most well-characterized anchor protein in vertebrates is CTCF, which mediates chromatin looping in concert with cohesin, probably through a loop-extrusion mechanism^2,35–39^. Considering our finding that MyoD apparently functions together with CTCF to regulate the insulation of CDBs, we questioned whether MyoD might control the formation of chromatin loops in muscle cells. Using the HiCCUPS algorithm^40^ to assess our BL-Hi-C dataset, we ultimately identified 24,492 chromatin loops in proliferating WT myoblasts and 31,243 chromatin loops in differentiating WT myocytes (Fig. 2a and Supplementary Data 2, Methods). The accuracy of the loop calls was supported by high-scoring aggregate peak analysis (APA) plots (Fig. 2a, Methods) and by observable enrichment of CTCF-bound loops in muscle cells (Supplementary Fig. 2a); of these loops, about 75% had convergent CTCF-binding motifs (Supplementary Fig. 2b).

We then searched for motifs enriched within accessible regions of the loop anchors, and detected significant enrichment for myogenic E-box motifs^28,29^ (Fig. 2b). This enrichment was confirmed by our observation that MyoD-binding peaks were enriched at loop anchors, as analyzed using pseudo-peaks (see Methods) on a combined MyoD ChIP-seq dataset generated from our present study and other published work^31^ (Fig. 2c, Supplementary Fig. 2c and Supplementary Data 3). We found that 44.18% of chromatin loops in GM cells were MyoD-bound (10.87% with both anchors bound by MyoD, 33.31% with one anchor bound by MyoD), while 43.93% of chromatin loops in DM cells were MyoD-bound (10.75% with both anchors bound by MyoD, 33.18% with one anchor bound by MyoD). Furthermore, the MyoD-binding peaks at loop anchors were concordant with the binding signals of CTCF and the cohesin complex subunit, SMC3^41^ (Supplementary Fig. 2d). We cataloged all of the identified chromatin loops into four types based on the binding of MyoD and/or CTCF at loop anchors in muscle cells: MyoD-MyoD(noCTCF), MyoD-MyoD(CTCF), MyoD-CTCF, and CTCF-CTCF (Fig. 2d, e). This classification was further supported by our ability to observe both colocalization of MyoD/CTCF and non-overlaid MyoD or CTCF signals under direct stochastic optical reconstruction microscopy (dSTORM), which is a single-molecule localization microscopy technique that can reveal the organization of specific proteins with a lateral resolution of ~20 nm^42^ (Fig. 2f). We quantified the colocalization of CTCF and MyoD signals on the binarized dSTORM images using cross-correlation analysis^43^ and pixel-by-pixel analysis (Supplementary Fig. 2e). We also found that the MyoD-bound loops were significantly shorter than the CTCF-CTCF loops (288 kb on average), and the MyoD-MyoD(noCTCF) chromatin loops were even shorter (113 kb on average) (Supplementary Fig. 2f). Together, these data substantiate the idea that MyoD is localized on the anchors of chromatin loops in muscle cells.

We next assessed whether MyoD could indeed mediate chromatin loop formation in vivo by investigating the effects of MyoD knockout on the MyoD-bound loops detected in WT cells. APA plots indicated that MyoD knockout significantly decreased the loop-strength values for both MyoD-bound chromatin loops (MyoD-MyoD(noCTCF), MyoD-MyoD(CTCF), and MyoD-CTCF loops) and CTCF-CTCF chromatin loops, and that these decreases were evident in both GM and DM cells (Fig. 2g and Supplementary Fig. 2g). These results support the idea that MyoD functionally contributes to the formation of both MyoD-bound and CTCF-bound chromatin loops in vivo.

### MyoD orchestrates chromatin loop formation in muscle cells

**Fig. 3:**
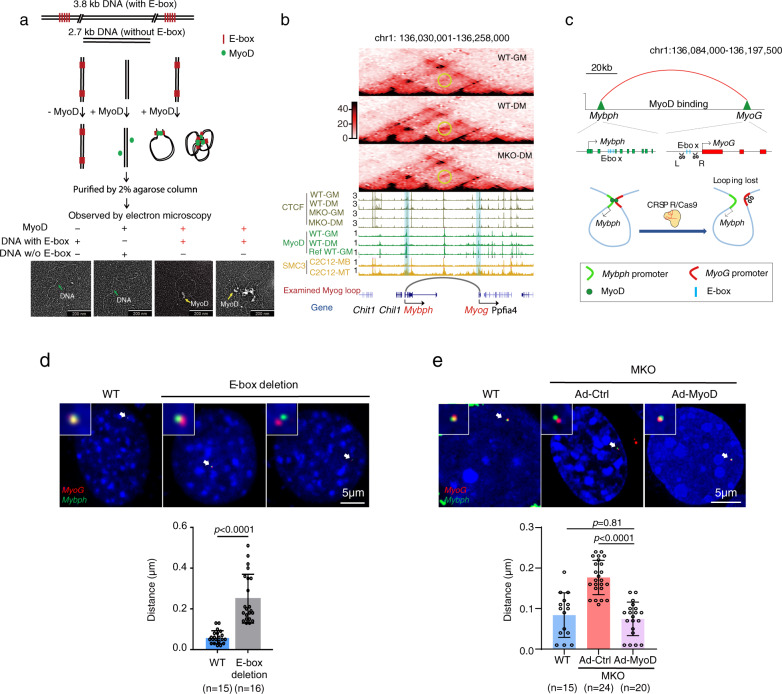
MyoD directly instructs chromatin looping dependent on the E-box. **a** MyoD mediates chromatin looping in vitro. The upper panel presents a schematic for the in vitro DNA circularization assay used to detect the ability of MyoD (green) to mediate DNA looping with E-box (red)-containing DNA (3.8 kb) and the no-E-box control (2.7 kb). The lower panel presents representative views of circularized DNA mediated by recombinant MyoD protein, as imaged by transmission electron microscopy (TEM). The images are representative of three independent experiments. **b** Hi-C contact map depicting normalized contact frequencies and ChIP-seq signal tracks for a MyoD-bound chromatin loop at the *MyoG* locus. The examined chromatin loop, which shows reduced Hi-C contacts in the MKO-DM sample compared with the WT-DM sample, is marked by a yellow circle. **c** Schematic showing CRISPR/Cas9-mediated deletion of the MyoD E-box in the regulatory region of the *MyoG* locus. **d** Representative images of 3D-DNA fluorescent in situ hybridization (3D-DNA FISH) in WT and E-box-deleted cells (described in **c**) cultured in DM. The upper panel shows that the *MyoG* promoter (red) and *Mybph* promoter (green) are separated by more in the E-box-deleted cells than in the WT cells. The lower panel showed the distance between the *MyoG-Mybph* loop anchors. The scale bar represents 5 μm. Data are presented as mean ± SEM. *p*-values were determined using unpaired two-tailed Student’s *t*-test, ***p < 0.001. Source data are provided as a Source Data file. **e** Representative dCas9-mediated DNA imaging (upper panel), which was used to visualize the chromatin loop anchors described in **b**. The distances between the two anchors were measured (lower panel). MKO cells were infected with adenovirus expressing MyoD (Ad-MyoD) or Ad-EGFP (control, Ad-Ctrl). The chromatin loopings at *MyoG* (red) and *Mybph* (green) loci were visualized by dCas9-mediated DNA imaging. DAPI served to visualize nuclei. The scale bar represents 5 μm. Data are presented as mean ± SEM. *p*-values were determined by performing ANOVA followed by Tukey’s multiple comparison test, ***p < 0.001. Source data are provided as a Source Data file.

Next, we examined whether MyoD directly mediates the formation of DNA looping in vitro and in cells. First, we performed an in vitro DNA circularization assay using a purified recombinant MyoD protein that was verified to have DNA-binding activity (Supplementary Fig. 3a) and a 3.8 kb linear DNA fragment containing five MyoD-binding sites at each end (Fig. 3a). The in vitro MyoD-mediated DNA looping was visualized by TEM. We found that MyoD did indeed facilitate linear DNA circularization in vitro in a MyoD-binding-motif-dependent manner (Fig. 3a and Supplementary Fig. 3b), indicating that MyoD can directly mediate DNA looping in vitro.

MyoD-instructed chromatin looping was further corroborated by deleting the MyoD-binding peaks from a MyoD-bound loop anchor. As shown in Fig. 3b, the *MyoG-Mybph* loop containing four E-boxes in the *MyoG* promotor and seven E-boxes in the *Mybph* promotor was a MyoD-mediated loop (Fig. 3b). We took this *MyoG-Mybph* locus as an example to test whether the E-boxes at the MyoD-loop anchor were required for MyoD-mediated looping in cells. To this end, we deleted about 410 bp containing four E-boxes of the *MyoG* promoter from the genome using the CRISPR strategy (Fig. 3c and Supplementary Fig. 3c). We then examined the formation of the MyoD-instructed loop at this mutant locus by Tn5-mediated fluorescence in situ hybridization (Tn5-FISH)^44^ (Fig. 3d). Consistent with the in vitro data, the MyoD-instructed loop was not detected at the E-box-deleted locus during muscle cell differentiation (Fig. 3d). Moreover, the expression of the cognate gene, *Mybph*, was significantly decreased in these cells (Supplementary Fig. 3d).

Finally, we took advantage of our observation that the MyoD-mediated *MyoG-Mybph* loop was lost in MyoD-null cells (Fig. 3b), and tested whether this lost MyoD-instructed loop could be re-established by reintroducing MyoD into MKO cells (Supplementary Fig. 3e). We visualized chromatin looping at the *MyoG* and *Mybph* locus by dCas9-mediated DNA imaging (Fig. 3e) and found that re-expression of MyoD (Ad-MyoD) was sufficient to re-establish the diminished loop at the *MyoG* and *Mybph* locus in MKO cells (Fig. 3e). This rescued loop formation efficiently induced transcription of *MyoG* and *Mybph*, as evidenced by the detection of nascent RNA products (Supplementary Fig. 3f). Collectively, these genetic, biochemical, and cellular imaging results demonstrate that MyoD functions as an anchor protein that orchestrates chromatin loop formation in muscle cells.

### MyoD anchors the formation of myogenesis-specific chromatin loops

**Fig. 4:**
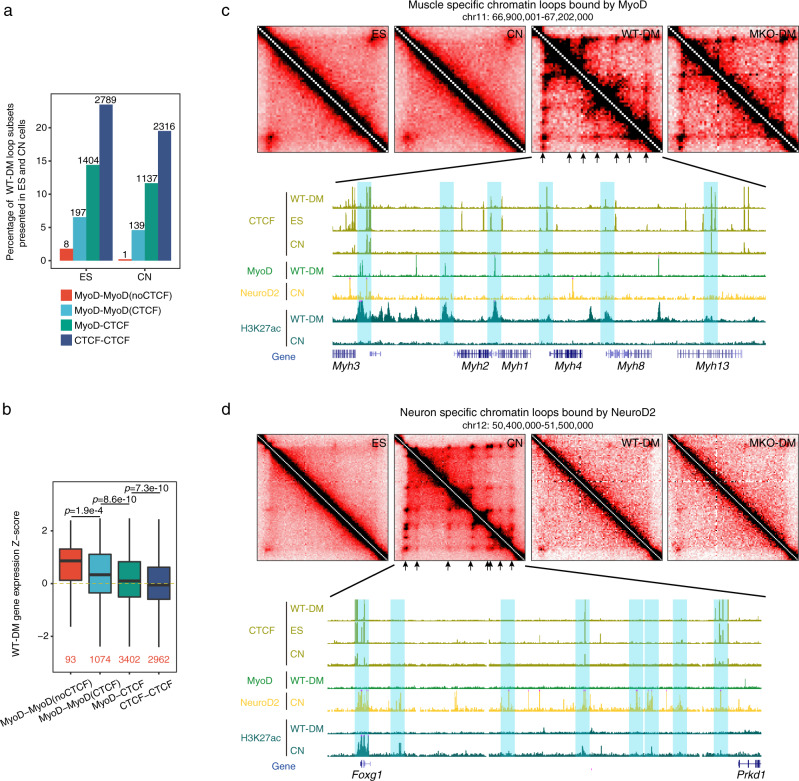
MyoD anchors the formation of myogenesis-specific chromatin loops. **a** Proportions of muscle chromatin loop subsets present in embryonic stem (ES) or cortical neurons (CN) cells. The numbers of muscle chromatin loops found in ES or CN cells are indicated for each bar. **b** Boxplots represent the expression Z-scores obtained in WT-DM cells for genes associated with each class of loops. The Z-score of each gene in WT-DM cells was calculated versus those in other ENCODE-collected cell types (ES, spleen, B, T, megakaryocyte, neural progenitor, and CN cells). The loop-related gene numbers are marked in red. *p*-values were calculated by the one-sided Wilcoxon rank-sum test. Source data are provided as a Source Data file. **c** Representative region showing muscle-cell-specific loops anchored on MyoD-binding regions at the *Myh* gene cluster. **d** Representative region showing neuronal cell-specific loops anchored on NeuroD2-binding regions at the *Foxg1* locus.

Based on the above findings, we next asked whether MyoD can mediate the organization of muscle-cell-specific chromatin looping. To test this, Hi-C data from embryonic stem (ES) and cortical neuron (CN) cells were collected^5^ and examined for the four types of chromatin loops identified in muscle cells. Relatively few MyoD-MyoD(noCTCF) loops were identified in ES cells (8 loops, 1.8%) or CN cells (1 loop, 0.2%) (Fig. 4a), revealing that the MyoD-MyoD(noCTCF) loops detected in muscle cells represent myogenic-lineage-specific chromatin loops. When we compared the expression tendencies of genes associated with the four loop types in WT-DM cells versus other cell types collected in the ENCODE database (see Methods), we found that the genes harboring MyoD-MyoD(noCTCF) loops exhibited the strongest extent of muscle-lineage-specific expression (Fig. 4b). Accordantly, the genes associated with MyoD-MyoD(noCTCF) chromatin loops were the most highly enriched for GO terms related to muscle cell differentiation (Supplementary Fig. 4).

To seek further molecular insights into MyoD-bound muscle-cell-specific chromatin looping, we examined ChIP-seq data for both MyoD and NeuroD2, which is a neuron-specific TF responsible for neuronal cell-lineage commitment and differentiation^32,45^, in both muscle and neuron cells. Only 18% of MyoD-binding peaks (34,881 pseudo-peaks) in muscle cells overlapped with NeuroD2-binding peaks (51,803 peaks) in cortical neuron cells, suggesting that MyoD and NeuroD2 might mediate the formation of separate sets of chromatin loops that can subsequently regulate distinct sets of target genes to properly specify lineage-specific cell identities. In support of this, we observed that the *Myh* gene cluster, which is located within a MyoD-bound muscle-cell-specific chromatin loop, was not detected in neuronal cells, which also lacked NeuroD2 binding at the same genomic region (Fig. 4c). Conversely, the chromatin loops bound by NeuroD2 at the *Foxg1* locus were found to be neuron-specific, and were not observed in muscle cells (Fig. 4d). Taken together, these results illustrate that cell-lineage-specific TFs may play genome-wide architectural roles in modulating 3D genome organization, and thus have a considerably larger functional impact than that held by the traditional concept that TFs affect lineage specification primarily by activating or regulating gene transcription.

### MyoD regulates primed architectural chromatin loops in proliferating muscle cells

Given previously published studies showing that MyoD constitutively binds to tens of thousands of sites genome-wide in proliferating cells^29,32^, together with our present findings that such binding only infrequently results in transcriptional activation at this stage and MyoD functions as an anchor protein for lineage-specific 3D genome organization, we sought to further explore the nature of MyoD’s genome-level architectural functions by reassessing our BL-Hi-C data. We were particularly interested in examining how the presence of MyoD influences internal interactions occurring within chromatin loops.

**Fig. 5:**
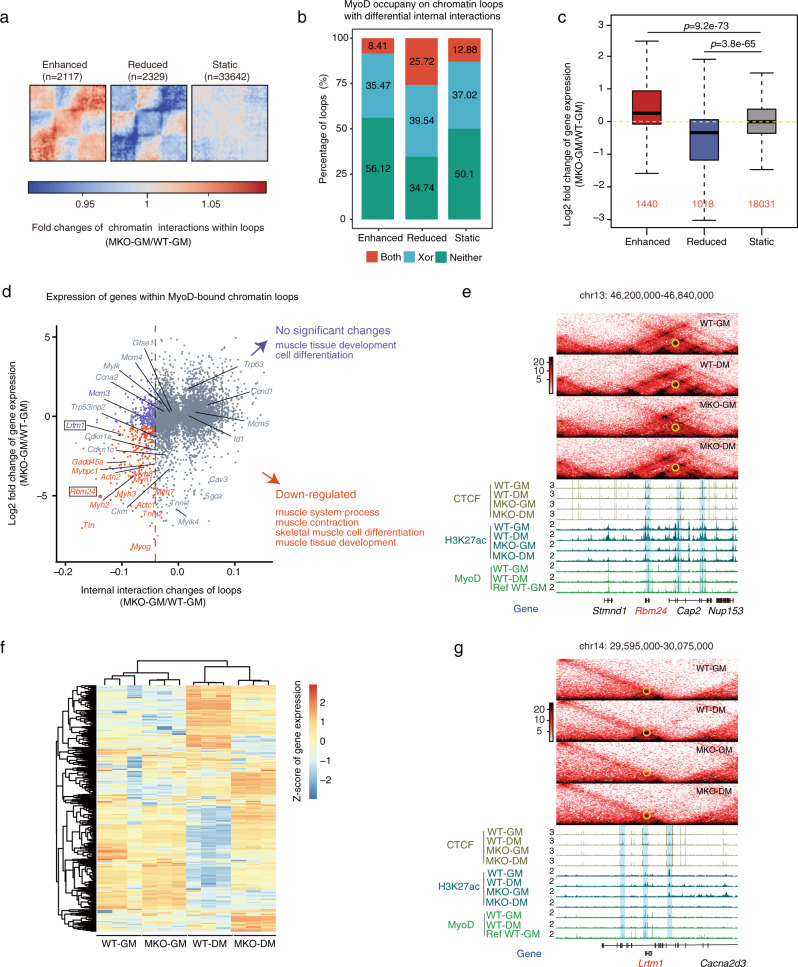
MyoD mediates primed architectural chromatin loops in proliferating muscle cells. **a** Aggregated Hi-C maps generated around chromatin loops with enhanced, reduced, or static internal interactions (measure by domain score) between WT and MKO myoblasts (MKO-GM vs. WT-GM). **b** Percentage of chromatin loops with differential internal interactions (MKO-GM vs. WT-GM) that are occupied by MyoD at anchors. MyoD bound at both anchors (Both, red), one of the two anchors (Xor, blue), or neither of the two anchors (Neither, green). Pseudo-peaks of MyoD binding identified from combined ChIP-seq data obtained from public datasets and our study were used for this analysis (see Methods). **c** Boxplot showing the corresponding expression changes of genes within chromatin loops having enhanced, reduced, or static internal interactions (domain score) identified in **b** (MKO-GM vs. WT-GM). Considering that a single loop may contain several genes and the domain scores of nested loops are associated with one another, we assigned each gene to its most dynamic nested loop. The loop domain-containing gene numbers are marked in red. *p*-values were calculated by the one-sided Wilcoxon rank-sum test. Source data are provided as a Source Data file. **d** Scatter plot showing the domain score fold changes and gene expression fold changes of MyoD-bound chromatin loops between WT-GM and MKO-GM cells. Internal interaction-reduced MyoD-bound chromatin loops whose associated genes did not show significantly differential expression in MKO-GM cells compared with WT-GM cells are shown as blue dots. In contrast, internal interaction-reduced MyoD-bound chromatin loops whose associated genes showed significant down-regulation in MKO-GM cells compared with WT-GM cells are shown as red dots. **e** The *Rbm24* locus as a representative internal interaction-reduced MyoD-bound chromatin loop associated with genes that were significantly downregulated in MKO-GM cells compared with WT-GM cells. **f** Heatmap showing expression (FPKM) Z-scores among the four indicated sample types for genes within internal interaction-reduced MyoD-bound chromatin loops whose associated genes did not show differential expression in MKO-GM cells compared with WT-GM cells. **g** The *Lmrt1* locus as a representative internal interaction-reduced MyoD-bound chromatin loop that is primed in proliferating muscle cells, comparing MKO-GM cells with WT-GM cells.

Previous studies indicated that CTCF and cohesin function together to establish architectural loops and that the extent of enhancer-promoter (E-P) interactions is elevated within such loops^46^. As our results revealed a notable colocalization of MyoD-binding peaks with CTCF (Supplementary Fig. 2d), we speculated that MyoD-bound loops might also feature increased intraloop interactions. To test this, we measured the internal interactions for each loop by calculating the “domain score” (D-score)^47^ in WT and MKO proliferating cells. MyoD binding was found to be enriched on the anchors of chromatin loops with reduced internal interactions (65%) in MKO-GM cells compared with WT-GM cells (Fig. 5a, b), indicating that MyoD regulates the internal interactions within chromatin loops by its direct binding on loop anchors, as also seen for CTCF and cohesin. Further analysis showed that interactions changes within chromatin loops were positively correlated with expression changes of the genes within those loops (Fig. 5c).

We next focused on analyzing the internal interaction changes of MyoD-bound loops and the expression changes of their cognate genes (Fig. 5d). We specifically examined MyoD-bound loops that displayed reduced interactions in MKO proliferating cells, because we hypothesized that these loops might be directly regulated by MyoD binding. We found that nearly 50% of the examined loops enclosed downregulated genes, including those exclusively regulated by MyoD in myoblasts and commonly regulated by MyoD in myoblasts and myocytes (Fig. 5d and Supplementary Fig. 5a–c). These genes were enriched for known myogenesis-related functions, indicating that interactions within these MyoD-bound loops are necessary for the regulation of gene expression related to muscle identity (Fig. 5d). For example, *Rbm24* is known to regulate myogenesis,^48^ and its differential expression in WT-GM and MKO-GM cells offers an excellent illustration of a gene that is enclosed within this type of MyoD-bound loop in muscle cells (Fig. 5e and Supplementary Fig. 5c, d).

Intriguingly, most (89%) of the remaining MyoD-bound loops with decreased interactions did not contain any gene that showed differential expression upon MyoD knockout (Fig. 5d). This finding underscores the apparent centrality of the architectural role (rather than the transcriptional activation role) of MyoD for these MyoD-bound loops in proliferating muscle cells. Although reduced interactions within these chromatin loops in MKO cells did not alter gene expression in proliferating cells, we found that genes within these chromatin loops were not turned on or off properly in MKO-DM cells compared to WT-DM cells (Fig. 5f), and this genetic dysregulation was clearly manifest in the defective differentiation phenotype of the MKO cells (Supplementary Fig. 1b)^20–22^. As the loops were still maintained in differentiated cells (Supplementary Fig. 5e), it appears that the interactions constrained within the loops in proliferating cells are effectively “primed” for muscle cell differentiation by the presence of MyoD, raising the possibility that some very early signal can somehow direct MyoD to occupy sites across the genome that subsequently permit both muscle-cell-appropriate loop architecture and later rewiring in response to differentiation signals. Supporting this idea, we found that *Lrtm1*, a myogenesis-related gene^49^ that is expressed only in differentiated muscle cells (Supplementary Fig. 5f), contains chromatin loops that have already been defined by MyoD in proliferating cells (Fig. 5g). Thus, mechanistically, our analyses of the MyoD-bound loops and their internal interactions reveal molecular insights into how MyoD exerts its architectural function, even in proliferating cells, to ensure the correct trajectory towards eventual muscle cell identity.

### The regulatory loops specified by MyoD are functionally required for muscle cell differentiation

**Fig. 6:**
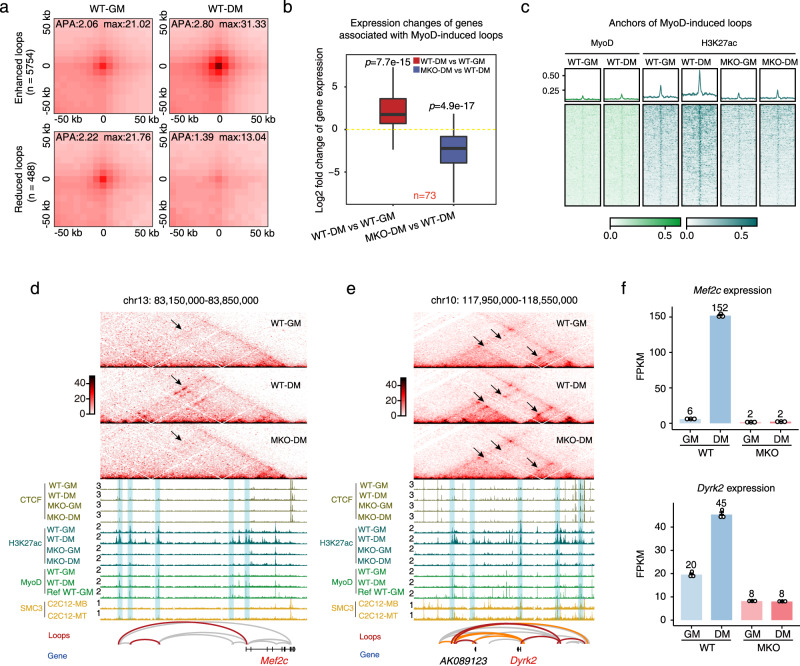
The regulatory loops specified by MyoD are functionally required for muscle cell differentiation. **a** APA plots showing the aggregated Hi-C contacts around chromatin loops that are enhanced (*n* = 5754) and reduced (*n* = 488) in WT-DM cells compared with WT-GM cells. Differential loops were identified based on DESeq2 (see Methods). **b** Boxplot showing expression changes of genes associated with MyoD-induced chromatin loops in WT-DM cells compared with WT-GM cells (left) and in MKO-DM cells compared with WT-DM cells (right). The gene numbers related to the MyoD-induced loops are marked in red. *p*-values were calculated by the one-sided Wilcoxon rank-sum test. Source data are provided as a Source Data file. **c** MyoD and H3K27ac ChIP-seq signals at the anchors of MyoD-induced loops. Heatmaps showing signal enrichment centralized at the anchors within a ± 100 kb genomic region. **d**, **e** The *Mef2c* and *Dyrk2* loci as representatives of MyoD-induced loops occurring during muscle cell differentiation. The MyoD-induced loops detected by our algorithm are presented in red in the loop track, while differentiation-induced loops are shown in orange. Other loops are presented in gray. **f** *Mef2c* and *Dyrk2* gene expression among WT and MKO cells cultured in GM and DM. Data are presented as mean ± SD. *n* = 3 biologically independent samples.

The genome is not only structurally organized within the nucleus but is also dynamically orchestrated in response to various cellular signals^5,47,50–53^. The biological function of MyoD in regulating muscle cell differentiation has been well documented, and very recent work showed that MyoD’s transcriptional activity is regulated by its ability to mediate chromatin interactions during *trans*-differentiation^54^. However, it remains largely unknown how MyoD impacts chromatin conformation dynamics during muscle cell differentiation. Using a modified differential loop detection method^53^ (see Methods), we identified 6242 differential chromatin loops (~25%) between proliferating and differentiating muscle cells. Among these loops, 5754 were significantly enhanced whereas 488 were reduced in differentiating cells (Fig. 6a). We detected significant enrichment for muscle cell differentiation-related genes among those associated with the enhanced chromatin loops (Supplementary Fig. 6a). These data also revealed that nearly 25% of chromatin loops that undergo dynamic changes during muscle cell differentiation contribute to 3D genome rewiring in a manner that functionally regulates myogenic differentiation.

A similar analysis performed on MKO cells identified 585 differential chromatin loops between WT-DM and MKO-DM cells. Consistent with the proposed regulatory role of these loops, 81.5% (477/585) were significantly reduced in the differentiated MKO cells (Supplementary Fig. 6b). Moreover, 71.57% (341/477) of the loops reduced in differentiated MKO cells had MyoD-binding peaks, supporting them as bona fide MyoD-mediated chromatin loops in differentiated WT cells (Supplementary Fig. 6c). Among the loops found to be enhanced under differentiation, 285 loops were bound by MyoD and disappeared when MyoD was absent from differentiating muscle cells (Supplementary Data 4), suggesting that they might be directly induced by MyoD. The genes associated with these MyoD-induced loops were significantly upregulated during differentiation and downregulated when MyoD was absent, verifying the regulatory role of MyoD-induced loops (Fig. 6b). In addition, acute depletion of MyoD with a Cre-mediated knockout system (MyoD^fl/fl^-Cre) yielded similar results: MyoD depletion significantly decreased the strength of these loops and the expression levels of their cognate genes during muscle cell differentiation (Supplementary Fig. 6d–l). Together, these findings show that MyoD-mediated loops are required for muscle cell differentiation.

Next, we sought to understand what drives the enhancement of MyoD-induced loops during muscle differentiation. We speculated that it might reflect an increase of MyoD binding on loop anchors during muscle cell differentiation. By examining MyoD-binding dynamics on anchors of MyoD-induced loops, we found that the majority of MyoD-induced loops did not show novel MyoD binding during the differentiation (Fig. 6c). However, we found that 88% of MyoD-induced loops were decorated with MyoD-dependent H3K27ac modification (Fig. 6c). Based on this observation, we speculated that H3K27ac might contribute to loop formation during muscle cell differentiation, and that MyoD might somehow actively direct the deposition of these modifications on the MyoD-induced loops.

Taking the *Mef2c* locus as a representative MyoD-induced loop, we sought to demonstrate its regulatory role in differentiation. We found that enhancer-promoter loops linked to *Mef2c* emerged during differentiation in a MyoD-dependent manner (Fig. 6d, Supplementary Fig. 6k). Concordantly, H3K27ac modification was enhanced in WT-DM cells but diminished in MKO-DM cells at this locus (Fig. 6d), and *Mef2c* gene expression was not induced in MKO-DM cells (Fig. 6f upper panel and Supplementary Fig. 6k). Similar results were obtained when we examined MyoD-induced loops within the *Dyrk2* locus (Fig. 6e and Supplementary Fig. 6l), from which the mRNA transcription product was only abundant in WT-DM cells (Fig. 6f lower panel and Supplementary Fig. 6l).

Collectively, these results demonstrate that MyoD orchestrates the formation of myogenic-lineage-specific chromatin loops that are required for the transcriptional regulation of myogenic genes during muscle cell differentiation.

### In MyoD-knockout cells, H3K27ac is insufficient to drive the formation of MyoD-bound chromatin loops

**Fig. 7:**
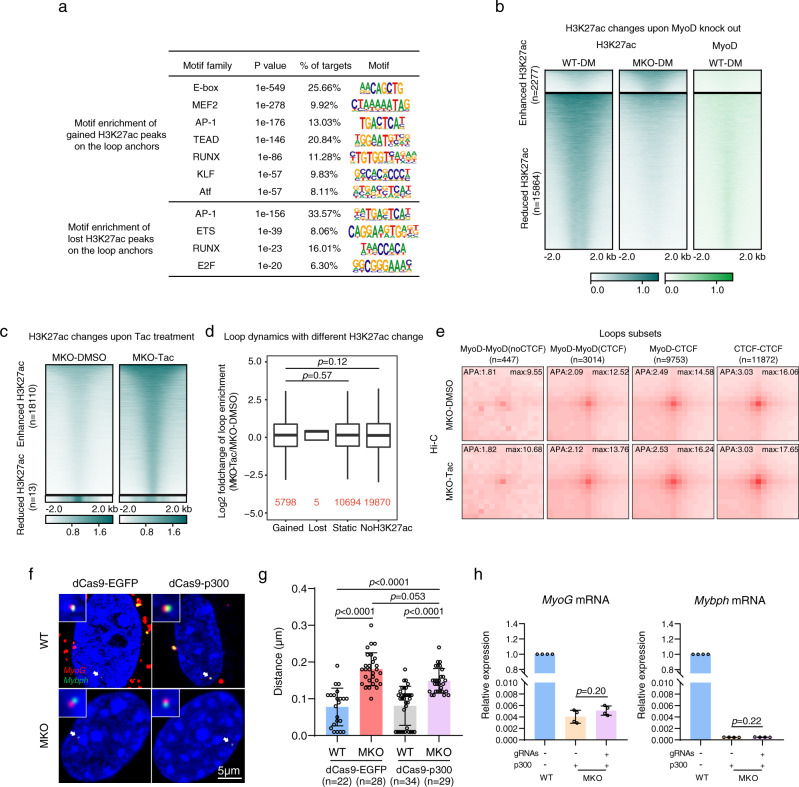
H3K27ac enhancement in MKO cells is not sufficient to drive the formation of MyoD-bound chromatin loops. **a** Motif enrichment at loop-anchor H3K27ac peaks gained or lost in WT-DM cells compared with WT-GM cells. *p*-values of motif enrichment were determined using cumulative binomial distributions, as applied by Homer. **b** Heatmaps showing H3K27ac peaks found to be enhanced and reduced on ChIP-seq analysis of MKO-DM cells versus WT-DM cells. MyoD-binding peaks at those differential H3K27ac peaks are also shown. **c** Heatmaps showing H3K27ac peaks enhanced and reduced on ChIP-seq analysis of MKO cells treated with the selective class I HDAC inhibitor, Tacedinaline (Tac), in DM for 12 h. DMSO-treated MKO cells served as a control. **d** Loop-strength fold changes of the loops with gained, lost, static or no H3K27ac marks on their anchors between MKO cells treated with Tac or DMSO. Loop strength was defined as relative Hi-C contacts found at loop pixels compared with those at surrounding donut-shaped background pixels (see Methods). The loop numbers are marked in red. *p*-values were calculated by the one-sided Wilcoxon rank-sum test. Source data are provided as a Source Data file. **e** APA plots showing aggregated Hi-C contacts around the four types of chromatin loops (classified in 2**d**) in MKO cells treated with Tac or DMSO (control). **f** Representative dCas9-mediated DNA images visualizing the chromatin loops linking *MyoG* (red) and *Mybph* (green) in WT and MKO cells transfected with dCas9-p300core and cultured in DM for 24 h. dCas9-EGFP served as a control. **g** The distance between the two anchors visualized in **f**. The scale bar represents 5 μm. Data are presented as mean ± SEM. *p*-values were determined by ANOVA followed by Tukey’s multiple comparison test, ***p < 0.001. Source data are provided as a Source Data file. **h** Relative expression of *MyoG* and *Mybph* in the cells described in **f**, as determined by qRT-PCR. Data are presented as mean ± SEM. *p*-values were determined by ANOVA followed by Tukey’s multiple comparison test. *n* = 3 biologically independent samples. Source data are provided as a Source Data file.

The dynamic organization of the 3D genome is known to facilitate the sophisticated interplay between accessible versus inaccessible chromatin states and TF occupancy^47^, but the interdependent impacts (and likely feedback) of chromatin status on 3D genome organization have remained largely elusive. In addition to our observation of MyoD-dependent H3K27ac modification on MyoD-induced loops, we found that during muscle cell differentiation, the loop anchors with increased H3K27ac signals showed overall enrichment for consensus E-box sequences (the known target motif of the MyoD protein) (Fig. 7a). When MyoD was knocked out, H3K27ac modification was remarkably decreased on MyoD-binding regions (Fig. 7b), further supporting our contention that overall H3K27ac modification depends on MyoD during differentiation. As MyoD itself stably binds around many myogenic genes in proliferating muscle cells, MyoD-dependent H3K27ac modification correlates even more specifically with loop formation or disruption. These results and the previously reported positive correlation between H3K27ac and loops^52,55^ prompted us to examine whether the H3K27ac-mediated active chromatin state directly triggers the formation of MyoD-bound chromatin loops during muscle cell differentiation.

We tested this possibility by treating MKO cells with the histone deacetylase I specific inhibitor, Tacedinaline (Tac), to enrich their H3K27ac signal throughout muscle cell differentiation. We found that Tac treatment led to the accumulation of H3K27ac modification in 18,110 genomic regions, including the anchors of MyoD-induced loops (96/285) (Fig. 7c). Furthermore, analysis of BL-Hi-C data from the Tac-treated MKO and DMSO-treated control cells showed that, in the absence of MyoD, the accumulation of H3K27ac per se was not sufficient to trigger the formation of new loops or enhance the signals for MyoD-bound or CTCF-CTCF chromatin loops (Fig. 7d, e). Consistently, Tac treatment could not rescue the differentiation defect of *MyoD*-null cells (Supplementary Fig. 7a, b).

To further validate the above results at a specific MyoD-mediated loop, we specifically increased the H3K27Ac level at the *Myog-Mybph* locus in *MyoD*-null cells using the dCas9-p300 system^56^. Toward this end, we applied sgRNAs to guide the dCas9-p300-core to each anchor of the *MyoG-Mybph* loop in *MyoD*-null cells (Supplementary Fig. 7c). The p300-facilitated acetylation of H3K27 at anchors of *MyoG-Mybph* loop was confirmed by ChIP-qPCR (Supplementary Fig. 7d), and chromatin loop formation at the *MyoG*-*Mybph* locus was examined by dCas9-mediated DNA imaging (Fig. 7f). We observed that the increased levels of H3K27ac mediated by dCas9-p300-core were not able to rescue *MyoG*-*Mybph* loop formation or activate transcription of *MyoG* or *Mybph* in the *MyoD*-null cells (Fig. 7g, h). Cells isolated from WT mice were used as a control (Fig. 7f–h).

Together, our data support the notion that H3K27ac accumulation in *MyoD*-null cells is insufficient to reconstruct MyoD-mediated chromatin loops, and further indicate that the master TF, MyoD, is a critical genome organizer in establishing the unique 3D genome architecture of muscle cells.

## Discussion

Each cell type acquires a unique stage-specific organization of its 3D genome during development. The cell-type-specific organization of the 3D genome can be regarded as an emergent property that is mediated by the interplay between TFs and chromatin-associated proteins^57^. Based on this model, the components involved in regulating the emergent property of chromatin should be coordinately orchestrated by a genome organizer that instructs the specific organization of the 3D genome in each cell type at a specific developmental stage. In this report, we provide compelling evidence indicating that MyoD exerts a previously unappreciated function as a genome organizer in muscle stem cells, and thereby has broad functional impacts on genome structure beyond its known functions in activating gene expression during development. Mechanistically, we reveal internal interaction changes in MyoD-bound loops that suggest insights into the potential mechanism through which cell-lineage pioneer TFs ultimately specify the positions and nature of chromatin loops representing the cell-type-specific internal interactions that enable differentiation to yield the correct lineage-specific cell identity. Furthermore, we biochemically demonstrate that interaction between MyoD and the structural protein, CTCF, drives the formation of different types of loops, ultimately supporting a lineage-specific genetic program. Finally, our group and others demonstrate that *MyoD*-null myoblasts exhibit decreased levels of H3K27ac and H3K4me1 at MyoD-bound enhancers^58^, raising the possibility that H3K27ac might be implicated in chromatin looping. By directing the CRISPR-mediated site-specific accumulation of H3K27ac at MyoD-bound loop anchors, we show that MyoD-instructed chromatin looping is independent of H3K27ac levels in muscle cells. This finding emphasizes that the occupancy of a master TF protein can specify the genome structures that form the basis for proper cell-lineage differentiation.

It has been well documented that MyoD and Myf5synergistically regulate myogenic gene expression by binding their consensus E-box sequences genome-wide^18^. Mice with genetic deletion of either MyoD or Myf5 show apparently normal muscle development^19,59–61^, but myogenesis is completely absent from *MyoD;Myf5* double-KO mice^16^. Those results indicate that MyoD and Myf5 can functionally substitute for each other to maintain myogenesis during development. Given this, it is conceivable that Myf5 might compensate for MyoD function in *MyoD*-null cells. This could explain why we detected a small number of reduced chromatin loops in *MyoD*-null myoblasts cultured in GM and why, given that *Myf5* is significantly downregulated in differentiating myoblasts, we observed substantially fewer reduced MyoD-bound loops in *MyoD*-null cells cultured in DM. Therefore, it would be important to test whether Myf5 might also be an anchor protein that instructs higher-order chromatin structure for muscle cell identity. A recent report found that transcription-mediated supercoiling regulates genome folding and loop formation^62,63^. We also know that MyoD binds DNA as an inactive form in proliferating myoblasts^64^. Based on these data, it is possible that the small observed difference of chromatin loops between WT and *MyoD*-null myoblasts might reflect a lack of MyoD-regulated transcription in the myoblasts if the formation of MyoD-bound chromatin loops is mediated in a cohesin-dependent manner. Using our current data, we cannot exclude the possibility that the MyoD-mediated formation of chromatin loops occurs via either the transcriptional role of MyoD or compensation by other proteins, such as Myf5, in myoblasts. Further investigation is needed to elucidate the detailed molecular mechanism underlying the action of MyoD in orchestrating chromatin loop formation in muscle cells.

ATPase-dependent chromatin looping is a well-known model for CTCF/cohesin complex-directed large DNA interactions^46^. Recent studies showed that TFs can also orchestrate the formation of chromatin loops that play critical roles in regulating the expression of the target genes of these TFs in various cell types^9,47,65,66^. Although various working models have been proposed^7^, the molecular mechanism underlying TF-mediated chromatin looping is largely unknown. Phase separation, which is one of the models proposed to explain TF-mediated small enhancer-promoter interactions^67–69^, was supported by two recently published papers: Ahn et al. reported that phase-separated NUP98–HOXA9, a homeodomain-containing transcription factor chimera recurrently detected in leukemias, induces CTCF-independent chromatin loops that are enriched at proto-oncogenes^70^. Moreover, Wang et al. demonstrated that Oct4 regulates TAD structures and chromatin loops for cell fate transition via a phase-separation mechanism^71^. Therefore, it is conceivable that MyoD might also mediate chromatin looping via a phase-separation mechanism.

It will be interesting to determine which signals ultimately initiate MyoD’s loop specification layer of genetic regulation at the earliest possible stage of myogenic-lineage-specific cell fate determination. It was recently reported that quiescent muscle stem cells (also known as satellite cells, SCs) have relatively dense heterochromatin^72–75^ and depletion of the paired-box transcription factor, Pax7, significantly reduces heterochromatin condensation in rare surviving SCs^72,73^, indicating that Pax7 regulates heterochromatin structure in quiescent muscle cells. Pax7 also critically contributes to the ability of SCs to generate committed myogenic progenitors by directly regulating *Myf5* and *MyoD*^76–78^. We speculate that Pax7 might act as an upstream regulator of MyoD-instructed chromatin looping by upregulating MyoD expression in SCs during myogenic development.

To extend our insights beyond the MyoD-mediated architectural regulation of muscle cell fate, we performed similar preliminary experiments with the well-studied neuron-specific TF, NeuroD2, in neuronal cells. Our results suggest that NeuroD2a has a highly similar loop specification function that apparently underlies the neuron lineage-specific 3D genome architecture and impacts (downstream) the genetic programming of future cell fate. We are eager to see if other lineage-specific TFs in different developmental systems and/or organisms play a general role, similar to that reported herein for MyoD, as genome organizers that orchestrate the lineage-specific chromatin and 3D genome structures required for cell fate determination during development.

## Methods

### C2C12 cell culture and differentiation

Mouse C2C12 cells (ATCC, CRL-1772) were cultured in growth medium (GM) consisting of Dulbecco’s modified Eagle’s medium (Gibco, Cat.N: C11995500BT) supplemented with 4.5 g/L glucose, 10% fetal bovine serum (Ausbian, Cat.N: VS500T), and 1% penicillin and streptomycin at 37 °C in a 5% CO_2_ atmosphere. For the differentiation of C2C12 myoblasts, cells were transferred to Dulbecco’s modified Eagle’s medium containing 2% horse serum and 1% antibiotics, and then cultured for 24 h. C2C12 cells were grown to 80–90% confluence before the induction of differentiation.

### Mouse lines and animal care

*MyoD*-knockout (MKO) mice (#002523) were obtained from the Jackson Laboratory. The floxed*-MyoD* mice in the C57BL/6j background were generated by the Model Animal Research Center of Nanjing University. Mice were housed in a pathogen-free facility and had free access to water and standard rodent chow under the following conditions: 21 °C ambient temperature, 50–60% humidity, 12 h dark/light cycle. All animal procedures were approved by the Animal Ethics Committee of Peking Union Medical College, Beijing, China (ACUC-A01-2016-003).

### Primary myoblast isolation, culture, and differentiation

Primary myoblasts were isolated from hind-limb skeletal muscles of MKO, WT littermates, or floxed-*MyoD* (*MyoD*^fl/fl^) mice at 2–3 weeks old, minced, and digested in a mixture of type II collagenase and dispase. Cells were filtered from debris, centrifuged, and purified to eliminate fibroblasts by differential attachment for 2 × 10 min. The obtained cells were cultured in a growth medium (F-10 Ham’s medium supplemented with 20% fetal bovine serum, 10 ng/ml basic fibroblast growth factor, 1% antibiotics) on collagen-coated cell culture plates at 37 °C in 5% CO_2._ For the differentiation of primary myoblasts, cells were transferred to Dulbecco’s modified Eagle’s medium (Gibco, Cat.N: C11995500BT) containing 2% horse serum (Hyclone, Cat.N: SH30074.03) and 1% penicillin and streptomycin, and then cultured for 24 h. All cells were grown to 60–70% confluence before induction of differentiation. For H3K27ac accumulation experiments, MKO myoblasts were treated with 2.5 μM Tacedinaline (Selleck, Cat.N: CI994) in DM for 12 h to enrich H3K27ac. DMSO-treated MKO myoblasts grown in DM for 12 h served as a control. The cells were then collected for ChIP-seq and BL-Hi-C. For acute depletion of MyoD, myoblasts from *MyoD*^fl/fl^ mice were infected with adenovirus-Cre (ad-Cre) and adenovirus-EGFP as control (ad-Ctrl). Two days (48 h) post-infection, EGFP-positive cells were sorted. The sorted cells were cultured in DM for 24 h and collected for RNA-seq and BL-Hi-C analyses.

### Cell preparation for BL-Hi-C and library construction

BL-Hi-C library construction was performed as previously described^19^ with some modifications. In brief, primary myoblasts were treated with 2% formaldehyde (Sigma, Cat.N: F8775) for 15 min (room temperature, R.T.) for protein–protein and protein–DNA crosslinking, and the reaction was quenched by adding glycine solution (F.C., 0.2 M) for 5 min (R.T.). The cells were scraped, transferred, and centrifuged, and the collected cells were split into aliquots of 3–5 × 10^5^ cells. Cell pellets were frozen in liquid nitrogen and stored at −80 °C until use.

For library construction, the cells were suspended in 1 ml of 1% SDS lysis buffer (50 mM HEPES-KOH, 150 mM NaCl, 1 mM EDTA, 1% Triton X-100, 1% SDS) at R.T. for 15 min and centrifuged at 500 × *g* for 2 min. The pellet was washed with 1 ml 0.1% SDS lysis buffer (50 mM HEPES-KOH, 150 mM NaCl, 1 mM EDTA, 1% Triton X-100, 0.1% SDS) and centrifuged at 500 × *g* for 2 min. The supernatant was discarded, and the cells were incubated with 50 µl of 0.5% SDS for 30 min at 37 °C. The SDS was quenched by the addition of 145 µl H_2_O and 12.5 µl 20% Triton X-100, and the genome was digested into blunt-end fragments with 5 µl HaeIII (10 U/µl, NEB, Cat.N: R0108L) in 81.5 µl H_2_O, 10 µl 10 × NEB buffer 2, 2.5 µl 20% Triton X-100, and 1 µl BSA at 37 °C for at least 2 h. The blunt ends of the DNA fragments were treated with adenine and ligated with Bridge Linker containing biotin for 4 h at R.T. The unligated DNA fragments were digested with 2 µl 10 mM dATP and 5 µl Klenow fragment (3′–> 5′ exo-) (NEB, Cat.N: M0212L) at 37 °C for 40 min. The cells were pelleted and resuspended with 79 µl H_2_O, 2 µl Bridge Linker, 10 µl T4 DNA ligase buffer, 5 µl 20% Triton X-100, 1 µl BSA, and 5 µl T4 DNA ligase at R.T. for 4 h. The cells were spun down and resuspended with 88 µl H_2_O, 10 µl lambda exonuclease buffer, 1 µl lambda exonuclease, and 1 µl exonuclease I to remove unligated Bridge Linker. The cells were then digested with 2 µl 10% SDS, 5 µl 20 mg/ml proteinase K (Ambion, Cat.N: AM2546), and 4 µl 5 M NaCl at 65 °C for 6 h or overnight. The DNA was purified by phenol–chloroform (Solarbio, Cat.N: P1012-500 ml) extraction and ethanol precipitation and fragmented to an average length of 300 bp using an S220 Focused-ultrasonicator (Covaris, Cat.N: 520045). The biotin-labeled DNA fragments were pulled down using streptavidin-coated Dynabeads M280 (Thermo Fisher, Cat.N: 11205D). The supernatant was discarded and the sample was incubated with 78 µl H_2_O, 10 µl T4 DNA ligase buffer, 1 µl 10 mM dNTP, 5 µl T4 polynucleotide kinase (NEB, Cat.N: M0201L), 1 µl T4 DNA polymerase (NEB, Cat.N: M0203L) and 5 µl large (Klenow) fragment (NEB, Cat.N: M0210L) at R.T. for 30 min to repair the ends. The supernatant was discarded and the sample was incubated with 83 µl H_2_O, 10 µl NEB buffer 2, 2 µl 10 mM dATP, and 5 µl Klenow fragment (3′–> 5′ exo-) (NEB, Cat.N: M0212L) at 37 °C for 30 min for A-tailing. The supernatant was discarded and the sample was mixed with 7.6 µl H_2_O, 10 µl 2 × quick ligase buffer, 2 µl quick ligase, and 0.4 µl 20 µM Y-adaptor at R.T. for 30 min for sequencing adaptor ligation. The library was PCR amplified from the beads for 12–14 cycles. After purification with AMPure XP beads (Beckman, Germany, Cat.N: A63881), each library was sequenced as double-end 150-bp reads. The sequences of the Bridge Linker and Y-adaptor were obtained from the BL-Hi-C paper^33^.

### ChIP and library preparation

Primary myoblasts were fixed with 1% formaldehyde in F-10 Ham’s medium at R.T. for 10 min and quenched with glycine (F.C., 0.125 M) for 5 min at R.T. The cells were scraped, pelleted, frozen in liquid nitrogen, and stored at 80 °C until further use. For IP, we used the following: 1 × 10^6^ cells and 2.5 µg antibody for H3K27ac, 2.5 × 10^6^ cells and 5 µg antibody for CTCF, and 1 × 10^7^ cells and 5 µg antibody for MyoD. The cells were thawed on ice, resuspended in cold cell lysis buffer (10 mM Tris pH 8.0, 10 mM NaCl, 0.5% NP-40) and 1 × EDTA-free protease inhibitors. The cells were lysed for 15 min on ice with inversion every 2 min, and centrifuged. The nuclear pellet was resuspended with cold nuclear lysis buffer (50 mM Tris pH 8.1, 10 mM EDTA, 1% SDS) and 1 × protease inhibitors. Nuclei were lysed for 15 min on ice with inversion every 2 min, and then sonicated for 24 cycles of 30 s on/30 s off using a Bioruptor (Diagenode). After sonication, a 10 × volume of IP dilution buffer (20 mM Tris pH 8.0, 2 mM EDTA, 150 mM NaCl, 1% Triton X-100, protease inhibitors) was added, and the chromatin was precleared using 25 µl protein A/G magnetic Dynabeads (Thermo Fisher, Cat.N: 88802/03) per IP for 1 h at 4 °C with rotation. The chromatin was incubated with the antibody for 6 h or overnight at 4 °C with rotation. The following antibodies were used for ChIP: MyoD, sc32758; H3K27ac, ab4729; CTCF, CST, Cat.N: 2899 S. Meanwhile, 25 µl beads per IP were blocked with 1 ml PBS/BSA (1 × PBS and 5 mg/ml BSA-fraction V) at R.T. for 3 h or at 4 °C overnight. The beads were then added to the chromatin/antibody and incubated for 4 h at 4 °C with rotation. Next, the beads were washed twice with cold low-salt wash buffer (20 mM Tris pH 8.1, 2 mM EDTA, 150 mM NaCl, 1% Triton X-100, 0.1% SDS), twice with high-salt wash buffer (20 mM Tris pH 8.1, 2 mM EDTA, 500 mM NaCl, 1% Triton X-100, 0.1% SDS), twice with cold LiCl wash buffer (10 mM Tris pH 8.1, 1 mM EDTA, 250 mM LiCl, 1% NP-40, 1% sodium deoxycholate), and once with cold 1 × TE buffer (10 mM Tris pH 8.0, 1 mM EDTA). The DNA:protein complexes were eluted twice for 10 min at 70 °C in 150 µl elution buffer (100 mM NaHCO_3_, 1 mM EDTA, 1% SDS). The complexes were combined with 5 M NaCl, RNase A, and proteinase K and reverse crosslinked at 65 °C for 6 h or overnight. DNA was purified using phenol/chloroform extraction and ethanol precipitation. Library construction was performed using an NEBNext® Ultra™ II DNA Library Prep Kit for Illumina (#E7530L, NEB).

### Total RNA library preparation

Total RNA was extracted using TRIZOL (Life Technologies, Cat.N: 15596018). Library construction was performed by ANNOROAD and Novagene. Sequencing libraries were constructed using an NEBNext® Ultra^TM^ RNA Library Prep Kit for Illumina following the manufacturer’s recommendations, and index codes were added to attribute sequences to each sample.

### Real-time RT-PCR analyses

Total RNA was extracted from cells with TRIZOL reagent and reverse-transcribed (RT) using RevertAid reverse transcriptase (Thermo Scientific, EP0442). For measuring nascent or mature *MyoG* and *Mybph* mRNAs, quantitative PCR (qPCR) analyses were performed with the Soso-Fast qPCR Master Mix (Bio-Rad, 1725202) using an iQ5 Multicolor Real-Time PCR Detection System (Bio-Rad). *GAPDH* served as an internal control. All primers used for RT-qPCR are listed in Supplementary Data 5.

### Library quality control and sequencing

Before sequencing, the libraries were quantified by qPCR and the size distribution was assessed using an Agilent 2100 Bioanalyzer. Hi-C libraries were sequenced at 2 × 150 bp pair-end on the Illumina Nova seq S2 platform. ChIP-seq libraries were sequenced at 1 × 50 bp single-end on the Illumina Nextseq550/Nova seq S2 platforms. RNA-seq libraries were sequenced at 2 × 150 bp paired-end on the Illumina Nova seq S2/HiSeq X10 platforms. All sequencing was performed by ANNOROAD and Novagene.

### Recombinant MyoD purification

The PCR-amplified DNA fragment encoding full-length MyoD (residues 1–318) was cloned into a modified pET28a vector with an N-terminal His6-SUMO tag and Ulp1 protease site. The constructed expression vector, named pET28a-SUMO-MyoD-FL, was transformed into *Escherichia coli* strain BL21 (DE3) (Agilent Technologies, Cat.N: 200131). The cells were grown in LB medium supplemented with 50 mg/ml kanamycin at 37 °C until the OD600 reached 0.6–0.8, and then 0.2 mM isopropyl β-D-1-thiogalactopyranoside (J&K, Cat.N: 367-93-1) was added to induce protein expression and the cells were cultured overnight at 18 °C. The cells were harvested by centrifugation at 5000 rpm (Thermo Fisher Scientific) for 15 min. The pellet was resuspended in buffer containing 20 mM Tris-HCl pH 8.0, 500 mM NaCl, and 25 mM imidazole pH 8.0, lysed under high pressure with a cell crusher (JNBIO JN-02C), and further clarified by centrifugation at 17,000 rpm for 60 min at 4 °C (Beckman Coulter). The supernatant was loaded onto 5 ml HisTrap FF columns (GE Healthcare) pre-equilibrated with Buffer 1 (20 mM Tris-HCl pH 8.0, 500 mM NaCl, 25 mM imidazole pH 8.0). His6-SUMO-MyoD-full-length (FL) was eluted from the column using elution buffer (Buffer 2; 20 mM Tris-HCl pH 8.0, 500 mM NaCl, 500 mM imidazole pH 8.0) with a stage-wise gradient on ÄKTA™pure (GE Healthcare). His6-SUMO tags were cleaved by Ulp1 protease during dialysis against buffer S (20 mM Tris-HCl, pH 8.0, 500 mM NaCl) and removed by a second-step HisTrap FF column (GE Healthcare). The MyoD-FL protein present in the flow-through was diluted with precooled 20 mM Tris-HCl pH 8.0 to ensure a low NaCl concentration, and excess nucleic acids were removed using a HiTrap SP FF column (GE Healthcare) with buffer A containing 20 mM Tris-HCl pH 8.0 and 200 mM NaCl and buffer B containing 20 mM Tris-HCl pH 8.0 and 1 M NaCl. The eluted protein was concentrated by centrifugal ultrafiltration (Millipore Amicon Ultra, 10 K), and loaded onto a pre-equilibrated HiLoad Superdex 75 16/60 column (GE Healthcare) in buffer GF (20 mM Tris-HCl, pH 8.0, 500 mM NaCl, 2 mM DTT) for final purification. All steps were performed on ice or at a low temperature.

### In vitro DNA looping assay and transmission electron microscopy (TEM)

We first constructed plasmid pUC57-MyoD, which contains 10 MyoD-binding sites (E-box, CAGCTG) separated by ~2.8 kb of intervening DNA. The intervening DNA was chosen as regions without MyoD occupancy or MyoD-binding motifs based on ChIP-seq and motif distribution data from muscle cells. The MyoD-binding motif (CAGCTG) was validated with EMSA (our previous data). The plasmid was digested with EcoRI (NEB, R0101) and gel purified. For the ligation assay, the DNA and proteins were incubated in binding buffer (20 mM Tris 7.9, 50 mM NaCl, 1 mM EDTA) at 25 °C for 20 min; the DNA:protein ratio was 1:100, wherein the final concentration of DNA was 80 nM and the final concentration of protein was 8 μM. The DNA–protein complexes were purified by gravity-flow gel filtration (4 ml of 2% agarose, ABT E-01508S-2B) using TE buffer (10 mM Tris 7.9, 1 mM EDTA). Peak fractions of DNA–protein complexes were analyzed by TEM. Briefly, samples were fixed with 0.4% glutaraldehyde in TE buffer on ice for 30 min. Then, DNA–protein complexes were mixed with a buffer containing spermidine (final concentration, 2 mM) to enhance the absorption of chromatin to the grids. Samples were loaded to glow-discharged carbon-coated EM grids, which were incubated for 2 min and then blotted. The grids were washed stepwise in 20 ml baths of 0%, 25%, 50%, 75%, and 100% ethanol solution for 4 min each (at R.T.), air-dried, and then shadowed with tungsten at an angle of 10° with rotation. Finally, the samples were examined using an FEI Tecnai G2 Spirit 120-kV TEM. Micrographs are shown in reverse contrast.

### E-box deletion

E-box deletion at the *MyoG* promoter was performed using a CRISPR/Cas9-mediated DNA editing system. Briefly, two sgRNAs (L and R) targeting the *MyoG* promoter were designed (Supplementary Data 5) and cloned to a backbone vector carrying a codon-optimized version of Cas9 with EGFP (Cas9-px458). The resulting plasmid, which harbored the two sgRNAs, was named Cag9-sgRNA-L&R. For E-box deletion in muscle cells, C2C12 cells were transfected with Cag9-sgRNA-L&R and cultured for 48 h. The EGFP-positive cells were FACS-sorted and plated to a 12-well plate. When the cells reached 80% confluence, they were changed to DM for 24 h. Finally, the cells were genotyped by PCR using four pairs of primers (Supplementary Data 5) and subjected to measurement of chromatin loop formation by Tn5-mediated 3D-FISH. The EGFP-negative cells served as negative controls.

### DNA FISH

DNA FISH probes were constructed according to a previously published paper (Tn5-FISH)^44^. Briefly, the probe library was generated by PCR amplification and recovered using a DNA Cleanup kit (Zymo Research, Cat.N: D4014). The primer sequences for probe amplification are listed in Supplementary Data 5. The Tn5-FISH probes were amplified by a second PCR with fluorescence-tagged primers. The in situ hybridization procedure of Tn5-FISH was similar to that of traditional FISH, as previously described^79^. Briefly, cells were seeded on coverslips and fixed with 4% paraformaldehyde for 15 min, washed with PBS for 5 min, and permeabilized with 0.1% Triton X-100/saponin solution for 10 min. Next, the cells were washed with PBS for 5 min, incubated in 20% glycerol for 20 min, snap freeze-thawed three times with liquid nitrogen, air-dried, and washed with PBS. The cells were further treated with 0.1 M HCl, permeated with 0.5% Triton X-100/saponin, digested with 100 µg/mL RNase A for 30 min at 37 °C, and then balanced in 50% deionized formamide/2 × SSC solution for 30 min. Each Tn5-FISH probe (10 ng) was mixed with hybridization buffer and applied to the slide, the coverslip was placed on top, and the slide was incubated at 75 °C for 5 min and then overnight at 37 °C. The coverslips were washed with wash buffer, stained with DAPI, and then mounted on slides with AntiFade mounting medium (Solarbio, Cat.N: S2100). Microscopic imaging was performed on a Leica TCS SP8 STED equipped with the spectral flexibility of white-light laser (WLL) for excitation and an HC PL APO × 100/1.4 oil objective.

### Immunofluorescent staining

Cells were fixed with 4% paraformaldehyde in PBS, blocked in 3% BSA, and stained according to standard protocols using primary antibodies against MyoD (Santa Cruz, sc32758, 1:500), CTCF (Millipore, 07-729, 1:200), Pax7 (DSHB, 1:15), MyoG (DSHB, F5D, 1:500), and MHC (DSHB, MF-20, 1:500). As secondary antibodies (for all but the dSTORM experiments), goat anti-mouse Alexa Fluor 594 (Zhongshanjinqiao Corporation, Cat.N: ZF0513, 1:200) was used for Pax7 and goat anti-mouse Alexa Fluor 488 was used for MyoD and MHC (Zhongshanjinqiao Corporation, Cat.N: ZF0512, 1:200). The secondary antibodies used for dSTORM were goat anti-mouse Alexa Fluor 488 (Life Technologies, Cat.N: A-11017, 1:1000) and goat anti-rabbit Alexa Fluor 647 (Life Technologies, Cat.N: A-21246, 1:1000).

### Western blot analysis

The cells were lysed in a buffer containing 50 mM Tris pH 7.5, 150 mM NaCl, 0.5% Nonidet P40, and protease and phosphatase inhibitors. Proteins in lysates were resolved by SDS-PAGE and transferred to a polyvinylidenedifluoride membrane. Immunoblotting was performed using primary antibodies against MHC (DSHB, MF-20, 1:500); MyoD (Santa Cruz, sc32758, 1:500) and β-tubulin (CMCTAG, AT0003, 1:1000). Membranes were washed for 30 min, incubated with HRP-conjugated secondary antibodies (Zhongshanjinqiao Corporation, Cat.N: ZB-2305, 1:2000) for 1 h at room temperature, and then washed with Tris-buffered saline containing 0.1% Tween-20 for 30 min. Membranes were then placed in detection solution (Thermo Fisher Cat.N: 34580), incubated for 1 min at room temperature, and subsequently exposed to a chemiluminescence instrument (Tanon, #5800). For full scans of all Western blots, see the Source Data file.

### Direct stochastic optical reconstruction microscopy (dSTORM)

Coverslips (Warner Instruments, 25 mm, #1.5 thickness) were used. C2C12 cells were cultured on cleaned coverslips coated with fibronectin (2 μg/mL in PBS, pH 7.4; Sigma). After cells were immunostained for MyoD and CTCF (see Immunofluorescent staining section above), they were blocked twice with 1 × PBS solution containing 50 mM glycine for 10 min, washed once in 1 × PBS, and then subjected to dual-color imaging in dSTORM imaging buffer (1 × PBS, 100 mM β-mercaptoethylamine (Sigma, Cat.N: 30070), pH 7.4) containing TetraSpeck beads (Life Technologies, Cat.N: T7279), which were used as fiducial markers for x−y drift correction and for overlaying the two-color images.

dSTORM images were acquired on an Olympus IX83 motorized inverted fluorescence microscope equipped with the CellTIRF-4Line system, a 150 × 1.45 NA total internal reflection objective, a back-illuminated EMCCD camera (Andor), an IX3-U-m4TIRSbx filter set (Olympus), and 488 nm (150 mW) and 640 nm (140 mW) lasers. dSTORM images of Alexa 647 and Alexa 488 were acquired sequentially in that order. All images were acquired at 10 frames per second. Individual molecules were localized and super-resolution images were reconstructed using the ThunderSTORM analysis module available for ImageJ.

### dSTORM colocalization analysis

Colocalization between MyoD and CTCF signals was quantified from the reconstructed dSTORM images^43^. Specifically, a pair of binarized MyoD and CTCF dSTORM images acquired from the same cell (described above) were first aligned based on the x−y coordinates of TetraSpeck beads appearing in both channels. Cross-correlation analysis and pixel-by-pixel calculation of the colocalization ratio were then performed on randomly selected, equally sized sections (regions of interest, ROIs) of these images.

Cross-correlation analyses of the dSTORM images were performed as previously described^43^. In brief, the cross-correlation function ${{{{{\rm{c}}}}}}(\vec{{{{{{\rm{r}}}}}}})$ between MyoD and CTCF signals within ROIs were computed in MATLAB using Fast Fourier Transforms (FFT) as follows:

$${{{{{\rm{c}}}}}}(\vec{{{{{{\rm{r}}}}}}})={{{{{\rm{Real}}}}}}\left(\frac{{{FFT}}^{-1}({FFT}({{Im}}^{{MyoD}})\times {{{{{{\mathrm{conj}}}}}}}({FFT}({{Im}}^{{CTCF}})))}{{\rho }^{{MyoD}}{\rho }^{{CTCF}}N(\vec{{{{{{\rm{r}}}}}}})}\right)$$

where *Im*^*MyoD*^ and *Im*^*CTCF*^ are input ROIs from binary dSTORM images of MyoD and CTCF proteins, *ρ*^*MyoD*^, and *ρ*^*CTCF*^ are the signal density within the ROIs, and $N(\vec{{{{{{\rm{r}}}}}}})$ is a normalization factor for the size of the ROIs.

ROIs from the MyoD and CTCF channels were overlaid and then subjected to pixel-by-pixel analysis of the colocalization ratio between the MyoD and CTCF signals using a custom-written MATLAB code. The percentage of MyoD signals that colocalized with CTCF signals (%MyoD colocalizing with CTCF) was calculated by dividing the number of pixels containing both MyoD and CTCF signals by the total number of pixels containing MyoD signals. The percentage of CTCF signals that colocalized with MyoD signals (%CTCF colocalizing with MyoD) was calculated by dividing the number of pixels containing both MyoD and CTCF signals by the total number of pixels containing CTCF signals.

### CRISPR-mediated DNA imaging

To visualize chromatin loop anchors, we took advantage of the CRISPR-mediated DNA labeling system^80,81^ and designed modifications to achieve non-repetitive DNA imaging. Briefly, 15 guide RNAs (crRNAs) targeting each anchor of the examined chromatin loop (*MyoG*-*Mybph* loop) were designed. The backbone sequences of the crRNA and tracrRNA were as previously described^80,81^. The sequences of crRNAs and tracrRNA used in this study are presented in Supplementary Data 5. The crRNAs were synthesized with fluorescent labeling at the 5′-end (Sango Biotech). The full-length tracrRNA was non-fluorescently synthesized by Integrated DNA Technologies (IDT). The crRNAs and tracrRNA were annealed and incubated with dCas9 protein (IDT) to form fluorescent RNA protein complexes (fRNPs) as previously described^81^. Primary myoblasts or C2C12 cells were pretreated with 100 ng/ml nocodazole (Sigma, M1404) for 16 h and transfected with the pre-assembled fRNPs or preannealed crRNA pool plus dCas9-p300core plasmid (gifts from Bo Huang) by electroporation using an SE Cell line 4D-Nucleofector™ X Kit (Lonza, Catalog#: V4XC-1024). The electroporated cells were plated in collagen-coated Nunc Glass Bottom Dishes (Thermo Fisher, 150680) and cultured for 12 h before imaging. Microscopic imaging was performed on a Leica TCS SP8 STED equipped with the spectral flexibility of WLL for excitation and an HC PL APO ×100/1.4 oil objective. Imaging was performed using maximum intensity projection with Z stacks from 0.2 to 3 μm. The optimal image processing was performed with STED-lighting. Some images were cropped to obtain key regions needed to measure the distance between DNA loci, with maximum values identified by Fiji (Image J)^82^ after maximum intensity projection. Nuclei were visualized using DAPI for fixed cells and Hoechst 33342 (Thermo Fisher, 62249) for living cells.

### RNA-seq data processing and differential gene expression analysis

Paired-end RNA-seq reads were obtained from three biological replicates of WT and MKO primary myoblasts at proliferation (GM) and early differentiation (DM) stages, with an average read depth of 4.5 × 10^7^ read pairs per sample. HISAT2 v2.1.0^83^ was used to align the reads to the mm9 genome, then HTSeq v0.6.0^84^ was applied to calculate the counts per gene under default parameters. Differential genes were identified with FDR < 0.01 and fold change > 1.5, using DESeq2 v1.24.0^85^. RNA-seq data from cells acutely depleted of MyoD with a Cre-mediated knockout system (MyoD^fl/fl^-Cre) and their controls were treated in the same way. Enrichment of GO terms among differential genes was assessed using DAVID v6.8.

### ChIP-seq data analysis

H3K27ac and CTCF ChIP-seq analyses were performed with two replicates in WT and MKO primary myoblasts at proliferation (GM) and early differentiation (DM) stages, while MyoD ChIP-seq analyses were performed in WT myoblasts and myocytes. H3K27ac ChIP-seq analyses were also performed in MKO cells with or without Tacedinaline treatment. ChIP-seq reads were mapped to the mm9 genome with Bowtie2 v2.3.5^86^ using default parameters. Aligned reads were filtered with a minimum MAPQ of 20, and duplicates were removed using SAMtools v1.9^87^. Peaks were called using MACS2 v2.2.5^88^ with default parameters for CTCF and MyoD. H3K27ac peaks were called by MACS2 using the broad-peak mode. Signal tracks were generated using the -SPMR option in MACS2. Then, the UCSC Genome Browser utility^89^, bedGraphToBigWig, was used to transform the bedgraph files to bigwig files. Differential peaks of ChIP-seq experiments were called with the R package, DiffBind v2.10.0^90^, under default settings. Heatmaps of ChIP-seq signal enrichment were generated by the Python package, deepTools v3.0.1^91^. ChIP-seq peak annotation was done by ChIPseeker v1.20.0^92^. The public ChIP-seq data used in this study were processed in the same manner. More specifically, we merged our detected MyoD peaks with those called from public MyoD ChIP-seq data (GSE56131) and determined the differential peaks by examining the MyoD-binding strength at merged peaks, comparing our WT-DM and WT-GM MyoD ChIP-seq data based on DiffBind v2.10.0. WT-GM pseudo-peaks combined static MyoD peaks and MyoD peaks with reduced signal in WT-DM, while WT-DM pseudo-peaks combined static MyoD peaks and MyoD peaks with enhanced signal in WT-DM cells.

### Motif enrichment analysis

The Homer^93^ script findMotifsGenome was used with default parameters to enrich for the motif of ATAC-seq peaks overlapped with loop anchors in WT primary myoblasts. The Homer script findPeaks was used with option –nfr to find nucleosome-free regions in the H3K27ac peaks. These nucleosome-free regions of the broad H3K27ac peaks were applied to call motifs, using findMotifsGenome with the size set to 200 bp.

### Hi-C data processing, Hi-C loop calling, and differential loop detection

BL-Hi-C data were processed for WT and MKO primary myoblasts at proliferation (GM) and early differentiation (DM) stages, MyoD^fl/fl^-Cre and MyoD^fl/fl^-Ctrl cells at the early differentiation stage, and MKO cells with or without Tacedinaline treatment. Non-redundant uniquely mapped contacts were generated utilizing an in-house HiCpipe pipeline, which trimmed the bridge linker, aligned reads, filtered artifact fragments, and removed duplicates. Juicer^40^ was used for Hi-C map generation, compartment partitioning, and loop calling. For every 100 kb bin, A or B compartments were defined by the over 70% replicates majority rule. Contact domain boundaries (CDBs) and their relative insulation scores were calculated by HiCDB, while insulation scores^94^ were calculated on MyoD- or CTCF-bound CDBs with the insulation score method (parameters: -is 1000000 -ids 200000 -im mean -nt 0.1). Differential CDBs were defined with a *t*-test FDR < 0.01 and a difference between cases and controls higher than the 80% quantile of the overall difference. The domain score^47^ (D-score) for each loop was calculated by dividing the intraloop interactions by all interactions connected to the corresponding loop. Loops with differential internal interactions (D-score) were determined with a *t*-test FDR < 0.01 and a fold change higher than the 80% quantile of the overall fold changes.

Loops were called under resolutions of 3, 5, and 10 kb with HiCCUPS using parameters (-p 6,4,2 -i 10,7,5) and scaled to 5 kb and 10 kb resolution. These parameters were also used to determine the loop strength of requested loops with HiCCUPS. Loop strength was calculated as relative Hi-C contacts at loop pixels compared with those at surrounding donut-shaped background pixels. The differential loop detection method was adapted from Douglas et al.^53^, and used DESeq2 to detect differential enrichment of the loop over background across conditions. To compare loops under two conditions, loops were first called on combined BL-Hi-C matrixes for each condition and merged together. Then, the loop pixels and their surrounding pixels were collected from each biological replicate at resolutions of 5 kb and 10 kb, according to the previous work^53^. To address a description of bias^52^ based on contact distance associated with the identification of differential interactions from Hi-C-like data, loops were split into distance regimes of greater or less than 150 kb for 5 kb and 10 kb resolution. Differential loops called from each set were thresholded by a FDR of 0.1 and combined to form the final set of dynamic loops. For dynamic loops detected under both 5 kb and 10 kb resolution, the 5-kb resolution loops were kept. Public in situ Hi-C data of in vivo embryonic stem (ES) and cortical neuron (CN) cells were processed by Hi-C-Pro v2.11.1^95^ in the mapping and filtering steps and then treated with the same processing framework as our BL-Hi-C data.

### Loop annotation, aggregation, and functional analysis

Loop anchors (scaled to 10 kb) were overlapped with MyoD- and CTCF-binding sites and classified into four classes using bedtools toolset v2.27.1^96^. MyoD or CTCF occupancy on loops was also determined by intersecting loop anchors and MyoD or CTCF peaks. Aggregate peak analysis (APA) plots were generated to assess the quality of loop detection and explore the characteristics of different loop classes, as applied by the Juicer APA command. The aggregated maps generated by Juicer were normalized by the actual supporting loop number and the *cis*-interaction pairs of each sample. The APA score was also calculated by the Juicer APA command as the ratio of Hi-C contacts at the central pixel versus the mean Hi-C contacts of the lower-left pixels. Enrichment plots for differential CDBs and loops with differential internal interactions were generated with coolpup.py^97^.To gain knowledge about the function of different loop types, we analyzed the loop anchors with GREAT v3.0.0^98^ using the association rule “single nearest gene and 100 kb max extension” to identify the enriched biological process. For loop internal interaction analysis, we examined the genes enclosed in the loops and assigned the genes to their most dynamic loops to obtain explicit gene-loop pairs. In other sections, genes associated with loops were determined if their promoters (±3 kb around the TSS) overlapped loop anchors. To measure the muscle-lineage specificity of genes associated with MyoD-bound and CTCF-bound loops, we calculated the Z-score of gene expression in WT-DM cells compared with those of other ENCODE-collected cell types (embryonic stem cells, spleen cells, B cells, T cells, megakaryocytes, neural progenitors, and cortical neuron cells). ClusterProfiler v3.12.0^99^ was used to enrich and compare GO terms for different sets of loops bound by CTCF or MyoD.

### Visualization

Tracks of Hi-C maps and ChIP-seq data were generated by pyGenomeTracks v3.1.2 and UCSC,respectively^100^. Hi-C maps of each condition were normalized by *cis*-interaction pairs.

### Statistics

Analysis-specific statistics were applied as described in each subsection. For comparisons of distributions, the Wilcoxon rank-sum test was employed in R. Data are represented as boxplots where the middle line is the median, the lower and upper hinges correspond to the first and third quartiles, respectively, the upper whisker extends from the hinge to the largest value no further than 1.5 × IQR from the hinge (where IQR is the interquartile range), and the lower whisker extends from the hinge to the smallest value at most 1.5 × IQR of the hinge. Data beyond the whiskers were ignored.

The statistical significance of two-group comparisons was calculated using the two-tail Student’s *t*-test and ANOVA followed by Tukey’s multiple comparison test. The following annotation applies for all figures: *p < 0.05, **p < 0.01, ***p < 0.001.

### Reporting summary

Further information on research design is available in the Nature Research Reporting Summary linked to this article.

## Supplementary information

Supplementary Information

Description of additional Supplementary File

Supplementary Dataset 1

Supplementary Dataset 2

Supplementary Dataset 3

Supplementary Dataset 4

Supplementary Dataset 5

Reporting Summary

## Data Availability

The data that support this study are available from the corresponding authors upon reasonable request. The raw sequence data reported in this paper have been deposited in the Genome Sequence Archive (GSA)^101^ under accession number CRA002490. The processed data of this paper have been deposited in the Gene Expression Omnibus (GEO) database under accession number GSE157339. Accession codes for the published data in GEO used in this study are as follows: MyoD ChIP-seq of WT primary myoblasts, GSE56131; MyoD ChIP-seq of WT primary myotubes, SRP001761; ATAC-seq of WT primary myoblasts, GSE63573; NeuroD2 ChIP-seq of embryonic cortical neuron cells, GSE67539; Hi-C data of mouse neural development as well as CTCF and H3K27ac ChIP-seq of embryonic cortical neuron cells, GSE96107; SMC3 ChIP-seq of C2C12 myoblasts and myotubes, GSE113248. Data were aligned to the mm9 genome. Source data are provided with this paper.

## Source data

Source Data

## Code availability

Custom scripts described in the Methods will be made available upon request.
