# Supplementary material for: MyoD is a 3D genome structure organizer for muscle cell identity
Source: Nat Commun. 2022 Jan 11;13:205. doi: 10.1038/s41467-021-27865-6 (PMC8752600; doi:10.1038/s41467-021-27865-6)
Supplement: Supplementary file 1 — Supplementary Information [file 41467_2021_27865_MOESM1_ESM.pdf]

Supplementary information for:

MyoD is a 3D genome structure organizer for muscle cell identity

Ruiting Wang[1,12], Fengling Chen[2,12], Qian Chen[1,12], Xin Wan[1,12], Minglei Shi[3], Antony K. Chen[4], Zhao Ma[4,5], Guohong Li[6,7], Min Wang[6,7], Yachen Ying[4,5], Qinyao Liu[1], Hu Li[1,8], Xu Zhang[9], Jinbiao Ma[10], Jiayun Zhong[10], Meihong Chen[1], Michael Q. Zhang[2,3,11]*, Yong Zhang[1]*, Yang Chen[1,2,3]* & Dahai Zhu[1,8]*

The supplementary information includes:

Supplementary Figure 1–7 and the figure legends

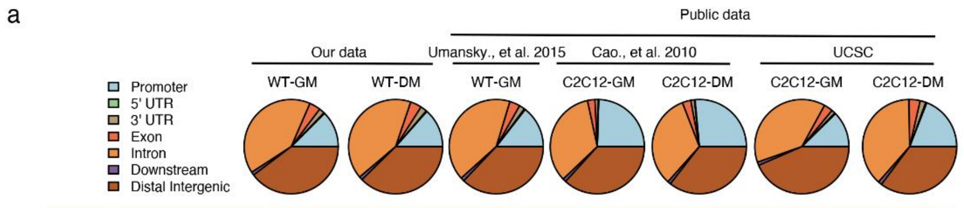

	WT-GM	WT-DM	WT-GM	C2C12-GM	C2C12-DM	C2C12-GM	C2C12-DM
Total	9258	9287	38843	27171	35408	13040	20160
Promoter	12.50	13.46	14.45	24.51	26.48	12.37	19.08
5'UTR	0.27	0.26	0.37	0.19	0.20	0.30	0.35
3'UTR	1.88	2.20	1.90	1.14	1.26	1.27	1.98
Exon	3.85	3.88	3.59	2.54	2.92	3.07	3.87
Intron	40.67	41.38	41.35	33.71	32.51	38.38	38.23
Downstream	1.12	1.16	1.24	1.13	1.08	1.12	1.29
Distal Intergenic	39.72	37.67	37.10	36.77	35.54	43.50	35.21

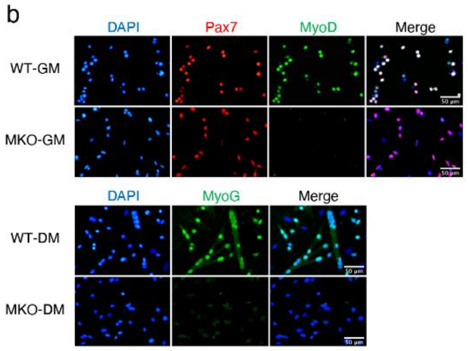

c

	library number	total PETs	valid pairs	valid pairs (rmdup)	cis interaction	cis rate
WT-GM	6	3,063,829,481	1,421,548,060	989,503,435	815,437,090	82%
WT-DM	8	3,398,918,116	1,507,933,582	1,080,862,988	808,788,438	75%
MKO-GM	6	2,526,066,578	1,303,369,549	732,185,200	641,222,613	88%
MKO-DM	8	3,201,103,857	1,427,826,726	971,256,657	714,955,332	74%

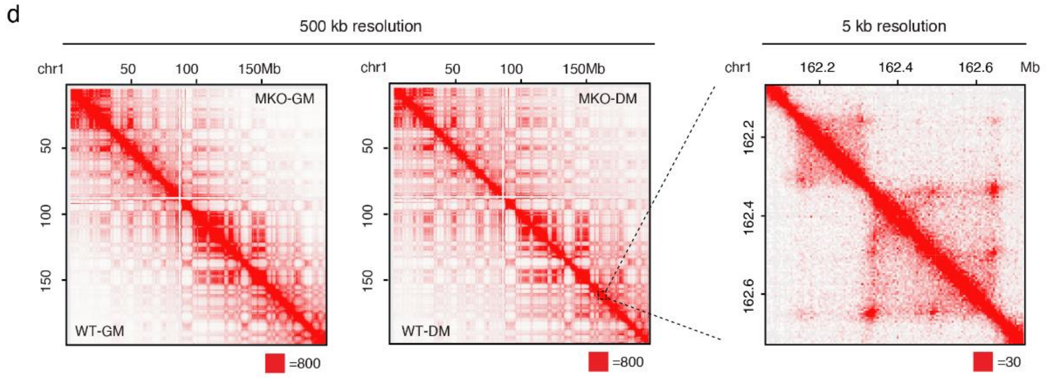

Supplementary Fig. 1| Unveiled genome architectural functions of MyoD in muscle cells. **a** Genomic distribution of MyoD binding peaks in proliferating or differentiating muscle cells. WT represents primary muscle cells while C2C12 is a skeletal muscle cell line. MyoD ChIP-seq data were from our study and public datasets of indicated references. Promoter is defined as ± 3 kb from the transcription start site (TSS). **b** Representative images for immunofluorescent staining of Pax7 (red), MyoD (green) and MyoG (green) in wild type (WT) and MyoD knockout (MKO) proliferating muscle stem cells (myoblasts, cultured in growth medium, GM) was shown at the upper panel. Corresponding data for differentiating

muscle stem cells (myocytes, induced to differentiation in differentiation medium, DM) was shown at the lower panel. DAPI (blue) served to visualize nucleus. The images are representatives of three independent experiments. Scale bars represent 50 μm. **c** Depth and quality of sequencing data for BL-Hi-C libraries on four types of samples (WT-GM, WT-DM, MKO-GM and MKO-DM). **d** Hi-C map showing BL-Hi-C data in our study reached 5 kb resolution. Left heatmaps showing chromatin contact matrices from the whole chromosome 1 (chr1) at 500 kb resolution in each of four cell samples. Right panel was a zoom-in heatmap for chr1: 162 ~ 162.8 Mb at 5 kb resolution. Maximum intensity was indicated in the lower right of each panel.

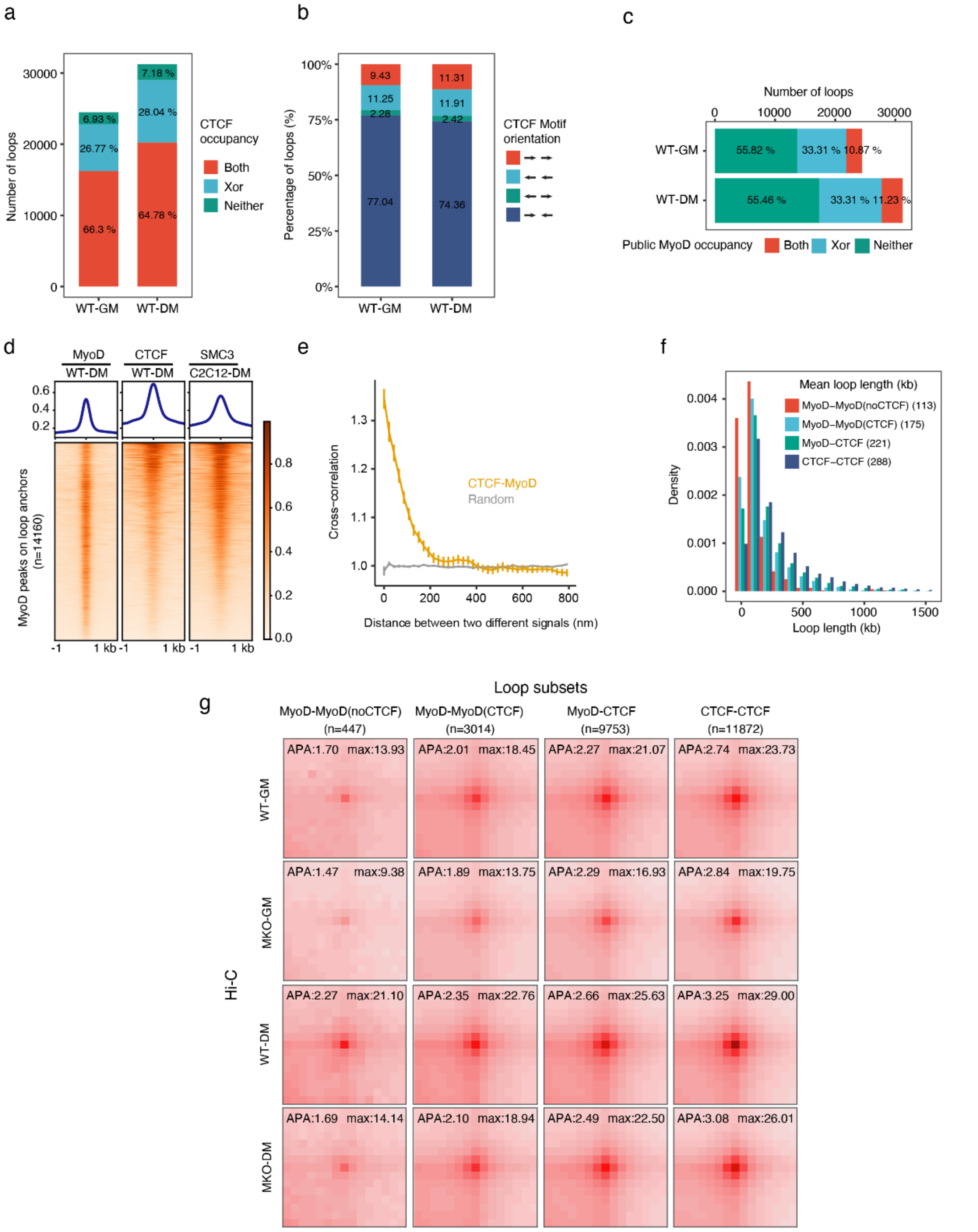

Supplementary Fig. 2| MyoD mediates chromatin loop formation *in vivo* and *in vitro*. a The percentages of chromatin loops with CTCF bound at both anchors (Both, red), one of two anchors (Xor, blue) or neither of two anchors (Neither, green) in WT-GM and WT-DM cells. **b** Distribution CTCF motif orientation on loop anchors bound by CTCF in WT-GM and WT-DM cells. **c** The percentage of chromatin loops with anchors overlapped by public MyoD

peaks in primary myoblast and primary myotube cells (GSE56131). **d** Heatmaps of ChIP-seq signal showing the enrichment of MyoD, CTCF and SMC3 on MyoD-bound loop anchors, indicating the concordant binding of MyoD with CTCF and SMC3 on these loop anchors. MyoD and CTCF ChIP-seq were conducted in our study. ChIP-seq for SMC3 in C2C12 cells were referenced from public database (GSE113248). **e** Quantification of CTCF and MyoD colocalization in the dSTORM images, determined by the average cross-correlation (C(r)). **f** Loop length distribution of MyoD-MyoD(noCTCF), MyoD-MyoD(CTCF), MyoD-CTCF and CTCF-CTCF chromatin loops. The average loop length was shown at upper right corner. **g** APA plots showing the aggregated Hi-C contacts around MyoD-bound three types of loops and CTCF-CTCF chromatin loops in MKO cells compared to WT cells. All four types of samples from GM and DM stages were shown. *n* represents number of each type of chromatin loops.

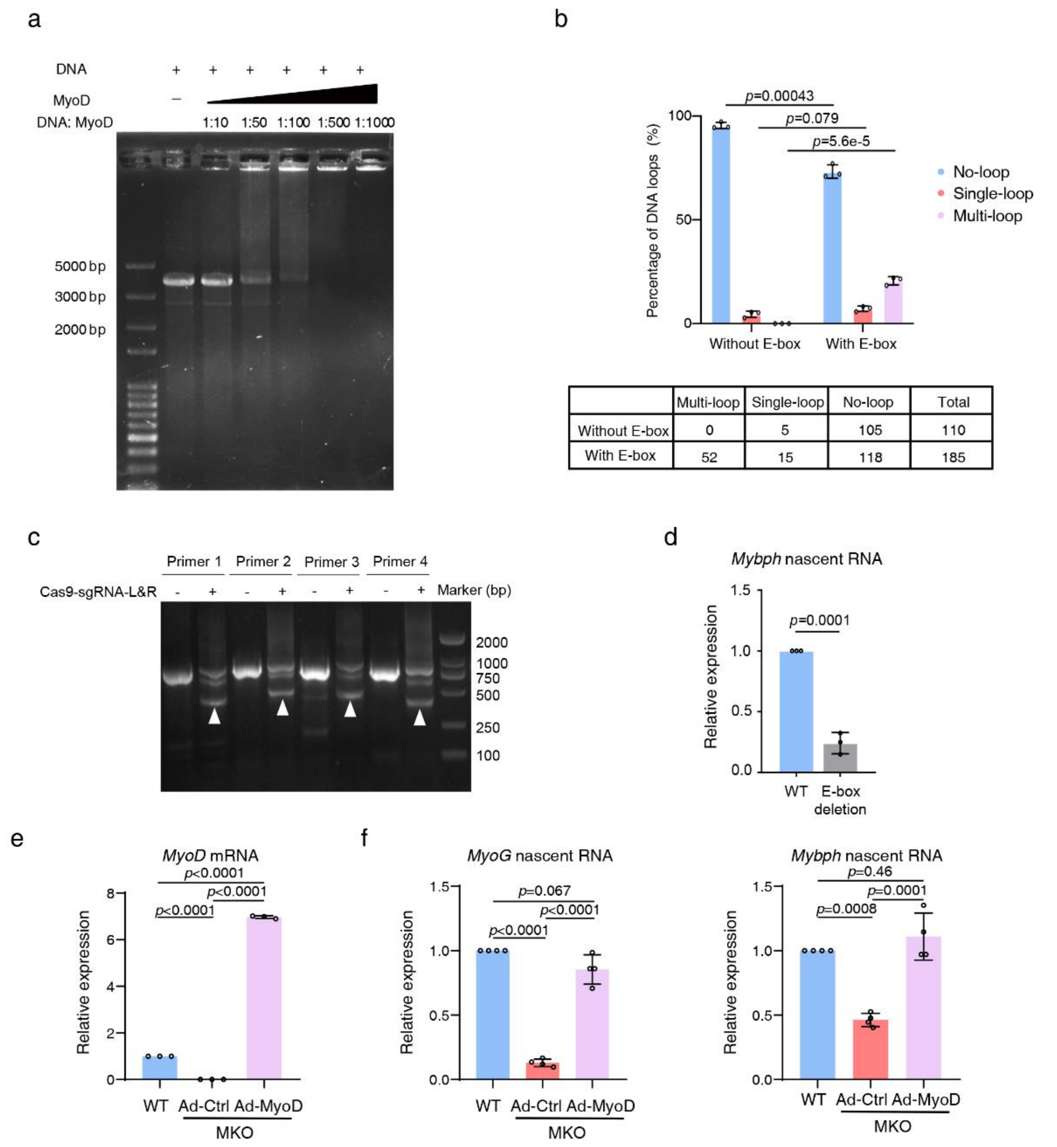

Supplementary Fig. 3| MyoD directly instructs chromatin loops. a Free and the recombinant MyoD protein-bound DNA fragment (3.7kb, with E-box) were shown and separated by polyacrylamide gel electrophoresis (PAGE). The binding of MyoD and DNA fragment also showed ratio-dependency. **b** Statistics of the percentage and number of loops observed with DNA circularization in Fig. 3**a**. Data are mean ± SD. *p*-values were determined using unpaired two-tailed Student's *t*-tests, $***p < 0.001$. $n = 3$ biologically independent experiments. Source data are provided as a Source Data file. **c** Genotyping of the wild-type (WT) and mutant (Cas9-sgRNA-L&R) cells, determined by PCR with four pairs of primers. The white triangles indicated the truncated bands. **d** Relative expression of the *Mybph* gene

was determined by examining nascent RNA with qRT-PCR. Data are mean ± SEM. *p*-values were determined using unpaired two-tailed Student's *t*-tests, ***$p < 0.001$. $n = 3$ biologically independent samples. Source data are provided as a Source Data file. **e** Relative expression of *MyoD* in the cells described in Fig. 3**e**, determined by qRT-PCR. Data are mean ± SEM. *p*-values were determined by performing an ANOVA followed by a Tukey's multiple comparison test, ***$p < 0.001$. $n = 3$ biologically independent samples. Source data are provided as a Source Data file. **f** Relative levels of nascent RNA of *MyoG* or *Mybph* in the cells described in Fig. 3e, determined by qRT-PCR. Data are mean ± SEM. *p*-values were determined by performing an ANOVA followed by a Tukey's multiple comparison test, ***$p < 0.001$. $n = 4$ biologically independent samples. Source data are provided as a Source Data file.

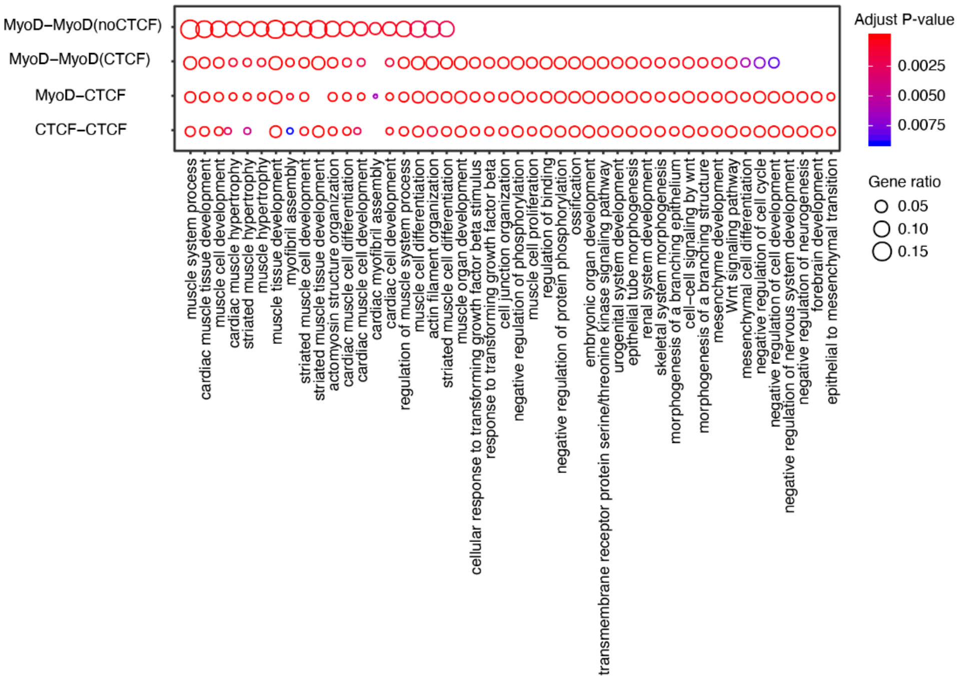

Supplementary Fig. 4| MyoD and NeuroD2 instructs lineage specific chromatin loops.

a The enriched GO terms of genes with promoter overlapped with anchors of MyoD-MyoD(noCTCF), MyoD-MyoD(CTCF), MyoD-CTCF and CTCF-CTCF chromatin loops. *p*-values of go term enrichment were determined using hypergeometric distribution and were adjusted by Benjamini–Hochberg method with ClusterProfiler.

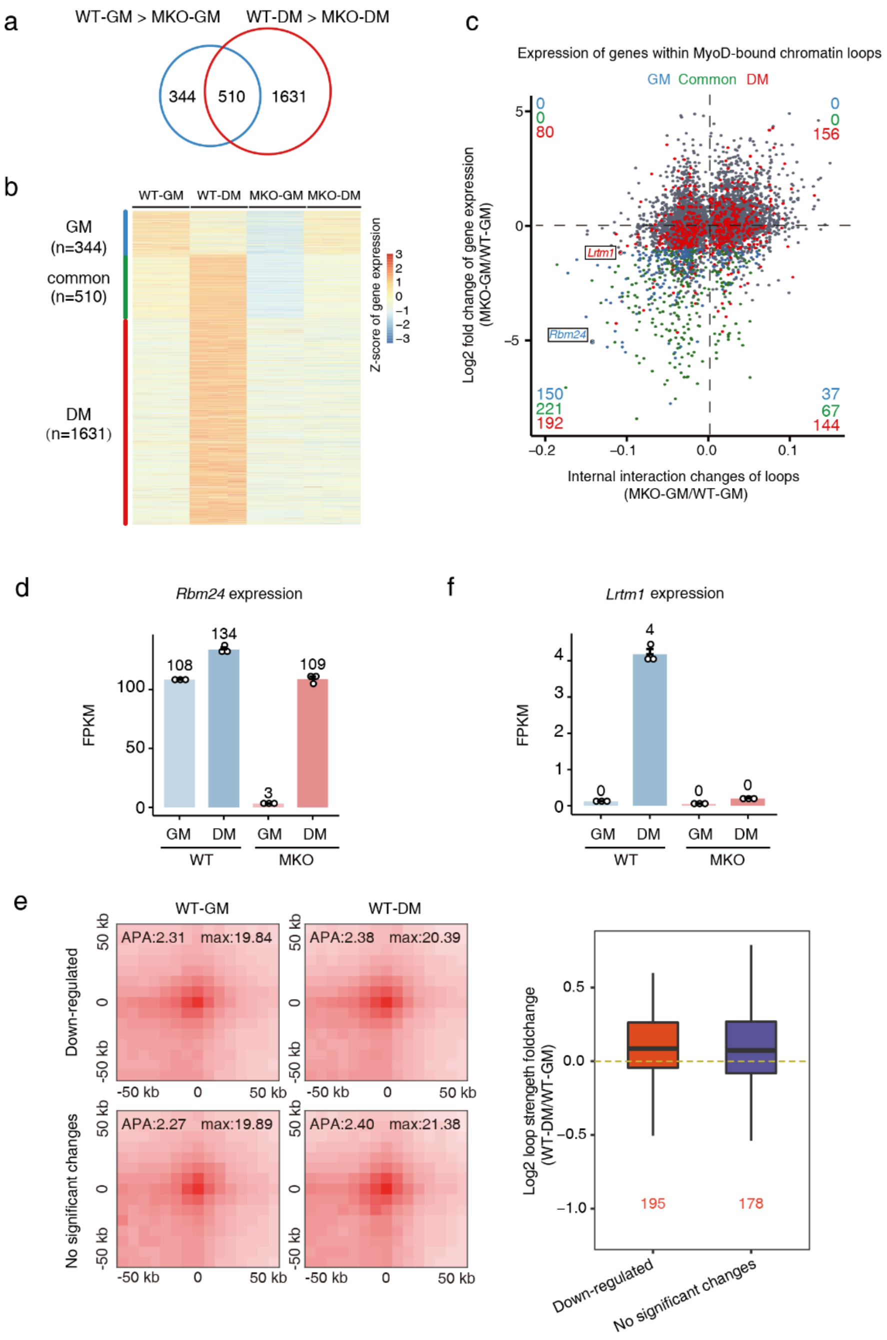

Supplementary Fig. 5| MyoD mediates primed architectural loops in myoblasts. a Venn plot of the genes regulated by MyoD in WT-GM and WT-DM. **b** Gene expression heatmap of MyoD regulated genes. **c** Scatter plot shows the gene expression fold changes versus the loop domain score fold changes between WT-GM and MKO-GM. The numbers of the genes commonly regulated or exclusively regulated by MyoD in WT-GM, WT-DM were marked in

each quadrant. **d** Boxplot showing the gene expression (FPKM) of *Rbm24* in four types of samples (WT-GM, WT-DM, MKO-GM and MKO-DM). Data are represented as mean ±SD. $n = 3$ biologically independent samples. **e** Loop changes of MyoD-bound architectural loops during differentiation. APA plots showing the aggregated Hi-C contacts around internal interaction reduced MyoD-bound chromatin loops with down-regulated genes or not significantly changed genes in WT-GM and WT-DM cells (left panel). Boxplot showed the loop strength fold changes for loops with down-regulated genes or not significantly changed genes between WT-DM and WT-GM cells (right panel). The loop numbers were marked in red. Source data are provided as a Source Data file. **f** Boxplot showing the gene expression (FPKM) of *Lrtm1* among four types of samples (WT-GM, WT-DM, MKO-GM and MKO-DM). Data are represented as mean ± SD. $n = 3$ biologically independent samples.

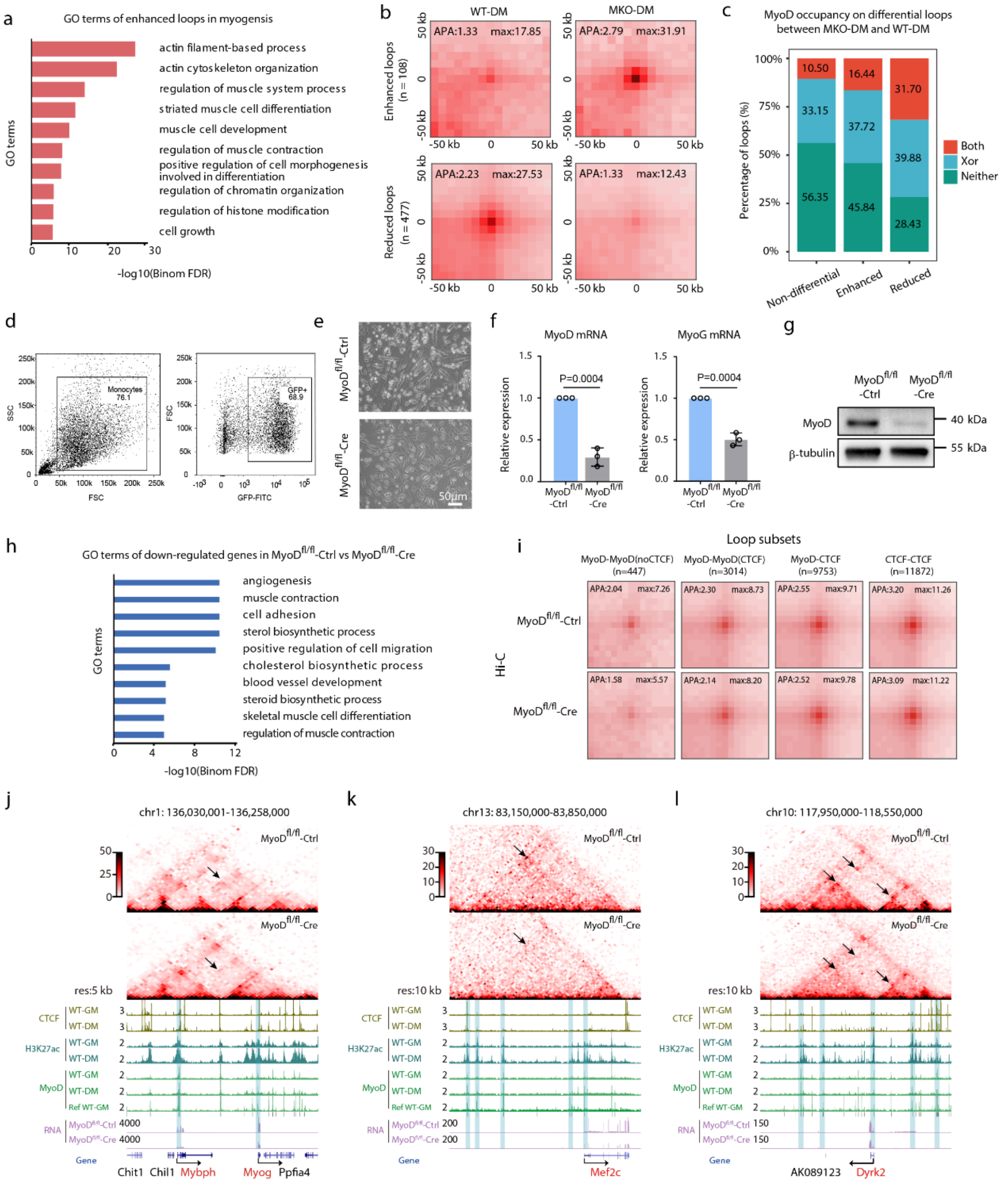

Supplementary Fig. 6| MyoD-induced regulatory loops are functionally required for muscle cell differentiation. a The enriched GO terms of enhanced loops in WT-DM cells compared with WT-GM cells. **b** APA plots showing aggregated Hi-C contacts around significantly reduced or enhanced chromatin loops in MKO-DM cells compared to WT-DM cells. *n* represents number of the differential chromatin loops between MKO-DM and WT-DM cells. Differential loops were identified with DESeq2 (Methods). **c** Percentages of MyoD-bound loops in the non-differential, enhanced or reduced chromatin loops described in

panel b. MyoD bound at both anchors (Both, red), one of two anchors (Xor, blue) or neither of two anchors (Neither, green) were shown. Pseudo-peaks of MyoD binding with combined ChIP-seq data from public and our study were used for this analysis. **d** Gating strategy used for FACS sorting of GFP positive cells. **e** Representative phase contrasts of primary myoblasts from MyoD$^{fl/fl}$ mice, infected with adenovirus expressing Cre enzyme (MyoD$^{fl/fl}$-Cre) and induced for differentiation 24 h. Infection with Ad-GFP served as control (MyoD$^{fl/fl}$-Ctrl). Scale bar represents 50 µm. **f** Relative expression of *MyoD* and *MyoG* genes in cells described in **e**, determined by qRT-PCR. Data are mean ± SEM. *p*-values were determined using unpaired two-tailed Student’s *t*-tests, $^{***}p < 0.001$. $n = 3$ biologically independent samples. Source data are provided as a Source Data file. **g** Representative immunoblot showing depleted MyoD protein in cells described in **e**. Source data are provided as a Source Data file. **h** Enriched GO terms of the differentially expressed genes between two type of cells described in **e**. **i** APA plots showing decreased strength of chromatin loops in MyoD$^{fl/fl}$-Cre compared to MyoD$^{fl/fl}$-Ctrl cells. **j-l** Hi-C heatmaps showing decreased loops at *MyoG-Mybph* (**j**), *Mef2c* (**k**) and *Dyrk2* (**l**) loci.

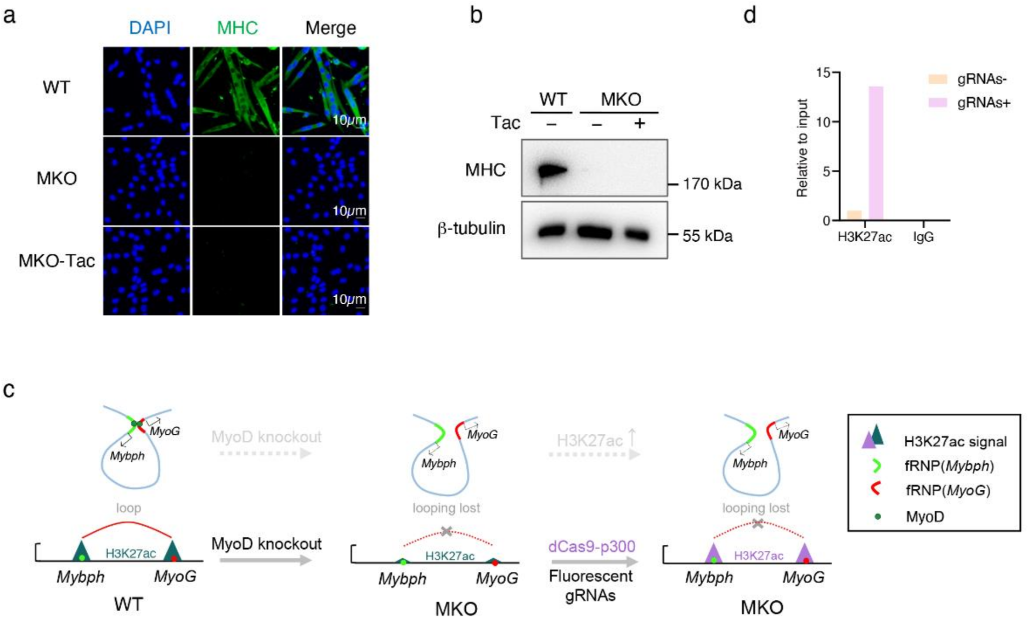

Supplementary Fig. 7| Accumulation of H3K27ac in MKO cells do not re-establish the diminished chromatin loop in *MyoD* null cells. a Representative images for immunostaining of myosin heavy chain (MHC, green) in WT or MKO cells treated with Tac in differentiation medium for 48 h. DAPI (blue) served to visualize nuclei. The images are representatives of three independent experiments. Scale bars represent 10 μm. **b** Representative western blot showing MHC protein in the cells described in **a**. β-tubulin served as equal loading control. The data are representatives of three independent experiments. Source data are provided as a Source Data file. **c** Schematic showing dCas9-p300-mediated site-specific accumulation of H3K27ac in MKO cells. **d** ChIP-qPCR with H3K27ac antibodies. The *MyoD* null cells were transfected with dCas9-p300core plasmid and sgRNA pool against each anchor of *MyoG-Mybph* loop in growth medium and subsequently subjected to ChIP analysis with H3K27ac antibodies. Mouse IgG served as control.
