# Supplementary material for: MyoD is a 3D genome structure organizer for muscle cell identity
Source: Nat Commun. 2022 Jan 11;13:205. doi: 10.1038/s41467-021-27865-6 (PMC8752600; doi:10.1038/s41467-021-27865-6)
Supplement: Supplementary file 2 — Description of additional Supplementary File [file 41467_2021_27865_MOESM2_ESM.pdf]

Description of additional Supplementary Data files

Supplementary Data 1: Gene expression and differential gene expression during differentiation and upon MyoD knock out.

Supplementary Data 2: Loops called by HiCCUPS in WT-GM, WT-DM, MKO-GM and MKO-DM cells.

Supplementary Data 3: MyoD pseudo-peaks in WT-GM and WTDM combining our data and data from Umansky., et al. 2015

Supplementary Data 4: Intersection of differential loops during differentiation and upon MyoD knock out.

Supplementary Data 5: Sequences of all primers, probes or sgRNAs.
