# Supplementary material for: MyoD is a 3D genome structure organizer for muscle cell identity
Source: Nat Commun. 2022 Jan 11;13:205. doi: 10.1038/s41467-021-27865-6 (PMC8752600; doi:10.1038/s41467-021-27865-6)
Supplement: Supplementary file 8 — Reporting Summary [file 41467_2021_27865_MOESM8_ESM.pdf]

nature research

Corresponding author(s): Dahai Zhu

Last updated by author(s): Dec 13, 2021

Reporting Summary

Nature Research wishes to improve the reproducibility of the work that we publish. This form provides structure for consistency and transparency in reporting. For further information on Nature Research policies, see our Editorial Policies and the Editorial Policy Checklist.

Statistics

For all statistical analyses, confirm that the following items are present in the figure legend, table legend, main text, or Methods section.

n/a	Confirmed	
☐	☒	The exact sample size (*n*) for each experimental group/condition, given as a discrete number and unit of measurement
☐	☒	A statement on whether measurements were taken from distinct samples or whether the same sample was measured repeatedly
☐	☒	The statistical test(s) used AND whether they are one- or two-sided<br>*Only common tests should be described solely by name; describe more complex techniques in the Methods section.*
☒	☐	A description of all covariates tested
☐	☒	A description of any assumptions or corrections, such as tests of normality and adjustment for multiple comparisons
☐	☒	A full description of the statistical parameters including central tendency (e.g. means) or other basic estimates (e.g. regression coefficient) AND variation (e.g. standard deviation) or associated estimates of uncertainty (e.g. confidence intervals)
☐	☒	For null hypothesis testing, the test statistic (e.g. *F*, *t*, *r*) with confidence intervals, effect sizes, degrees of freedom and *P* value noted<br>*Give P values as exact values whenever suitable.*
☒	☐	For Bayesian analysis, information on the choice of priors and Markov chain Monte Carlo settings
☐	☒	For hierarchical and complex designs, identification of the appropriate level for tests and full reporting of outcomes
☒	☐	Estimates of effect sizes (e.g. Cohen's *d*, Pearson's *r*), indicating how they were calculated

Our web collection on statistics for biologists contains articles on many of the points above.

Software and code

Policy information about availability of computer code

Data collection	No special software was used.
Data analysis	HISAT2 (v2.1.0), HTSeq (v0.6.0), DESeq2 (v1.24.0), DAVID (v6.8), Bowtie2 (v2.3.5), SAMtools (v1.9), MACS2 (v2.2.5), UCSC Genome Browser utility, DiffBind (v2.10.0), deeptools (v3.0.1), ChIPseeker (v1.20.0), homer, Juicebox (v1.9.8), HiCDB, insulation scores, HiC-Pro (v2.11.1), bedtools (v2.27.1), coolpup.py, ClusterProfiler (v3.12.0), GREAT (v3.0.0), pyGenomeTracks (v3.1.2), Matlab (2019a), ImageJ (2.0.0)<br>custom codes: HiCpipe (https://github.com/ChenFengling/HiCpipe)

For manuscripts utilizing custom algorithms or software that are central to the research but not yet described in published literature, software must be made available to editors and reviewers. We strongly encourage code deposition in a community repository (e.g. GitHub). See the Nature Research guidelines for submitting code & software for further information.

Data

Policy information about availability of data

All manuscripts must include a data availability statement. This statement should provide the following information, where applicable:

- Accession codes, unique identifiers, or web links for publicly available datasets
- A list of figures that have associated raw data
- A description of any restrictions on data availability

The raw sequence data reported in this paper have been deposited in the Genome Sequence Archive (GSA) under accession number CRA002490 that are publicly accessible at https://bigd.big.ac.cn/gsa. The processed data of this paper have been deposited in the Gene Expression Omnibus (GEO) database under the accession number: GSE157339.
Accession codes of the published data in GEO used in this study are as follows: MyoD ChIP-seq of wild-type primary myoblasts, GSE56131; MyoD ChIP-seq of wild-type primary myotubes, SRP001761; ATAC-seq of wild-type primary myoblasts, GSE63573; NeuroD2 ChIP-seq of embryonic cortical neuron cells, GSE67539; Hi-C

data of mouse neural development as well as CTCF and H3K27ac ChIP-seq of embryonic cortical neuron cells, GSE96107; SMC3 ChIP-seq of C2C12 myoblast and myotube, GSE113248. Data were aligned to mm9 genome. Source data are provided with this paper.

Field-specific reporting

Please select the one below that is the best fit for your research. If you are not sure, read the appropriate sections before making your selection.

☒ Life sciences ☐ Behavioural & social sciences ☐ Ecological, evolutionary & environmental sciences

For a reference copy of the document with all sections, see nature.com/documents/nr-reporting-summary-flat.pdf

Life sciences study design

All studies must disclose on these points even when the disclosure is negative.

Sample size	No statistical methods were used to predetermine sample size. Sample sizes for 2 biological replicates of MyoD, CTCF, H3K27ac ChIP-seq and 3 biological replicates of RNA-seq in this study were used according to common practice in the field. Sample sizes for 3 to 8 biological replicates of Hi-C were determined in order to get enough complexty and depth for loop analysis.
Data exclusions	There is no data that were excluded from the analyses.
Replication	Replication of sequencing data was confirmed by calculating correlation and reproducibility between replicates, as shown in figure and figure legend. Experiments were preformed 2 to 3 times. Similar observation was made for each replicate. Merged representative result was shown in the figures.
Randomization	Experimental materials were not divided into random subgroups. Most comparisons were done between determined stages, or WT and mutants.
Blinding	The investigators were not blinded to allocation during experiments and outcome assessment, as the information on genotype and developmental stage of materials was essential for the experiment design and analysis.

Reporting for specific materials, systems and methods

We require information from authors about some types of materials, experimental systems and methods used in many studies. Here, indicate whether each material, system or method listed is relevant to your study. If you are not sure if a list item applies to your research, read the appropriate section before selecting a response.

Materials & experimental systems

n/a	Involved in the study	
☐	☒	Antibodies
☐	☒	Eukaryotic cell lines
☒	☐	Palaeontology and archaeology
☐	☒	Animals and other organisms
☒	☐	Human research participants
☒	☐	Clinical data
☒	☐	Dual use research of concern

Methods

n/a	Involved in the study	
☐	☒	ChIP-seq
☐	☒	Flow cytometry
☒	☐	MRI-based neuroimaging

Antibodies

Antibodies used	MyoD antibody: Santa Cruz, sc32758, 5.8A; H3K27ac antibody: Abcam, ab4729; CTCF antibody: Cell Signaling, 2899S; CTCF antibody: Millipore, 07-729; Pax7 antibody: DSHB(no specified catalog number); MyoG antibody: DSHB, F5D; MHC antibody: DSHB, MF20; β-tubulin antibody: CMCTAG, AT0003; Goat anti-mouse Alexa Fluor 488 (Life Technologies, A-11017); goat anti-rabbit Alexa Fluor 647(Life Technologies A-21246) and HRP-conjugate goat Anti-mouse IgG (ZSGB-BIO, ZB-2305).
Validation	MyoD antibody (Santa Cruz, sc32758, 5.8A) is suitable for IF, western blot and ChIP-seq in mouse cells; H3K27ac antibody (Abcam, ab4729) and CTCF antibody (Cell Signaling, 2899S) are suitable for ChIP-seq in mouse cells; CTCF antibody (Millipore, 07-729), Pax7 antibody (DSHB), MyoG antibody (DSHB, F5D), and MHC antibody (DSHB, MF20) are suitable for IF in mouse cells. β-tubulin antibody (CMCTAG, AT0003) is suitable for western blot in mouse cells.

Eukaryotic cell lines

Policy information about cell lines

Cell line source(s)	Mouse C2C12 cells were from ATCC (CRL-1772).

Authentication	MyoD is expressed in proliferating and differentiating C2C12 cells. C2C12 cells can differentiate to myocytes and myotube with the sequencial expression of MyoG and MHC, measured by quantitavie RT-PCR, western blot and immunofluorescent staining.
Mycoplasma contamination	All cell lines tested negative for mycoplasma contamination.
Commonly misidentified lines (See ICLAC register)	None

Animals and other organisms

Policy information about studies involving animals; ARRIVE guidelines recommended for reporting animal research

Laboratory animals	Mus musculus, strain: MyoD knockout mice (Jax #002523) were backcrossed with C57BL/6j for 2 generations (N2) in our colony and floxed MyoD mice with C57BL/6j background, male and female, 2~3 weeks.
Wild animals	None
Field-collected samples	None
Ethics oversight	All the animal procedures were approved by the Animal Ethics Committee of Peking Union Medical College, Beijing, China (ACUC-A01-2016-003). Mice were housed in the animal facility and had free access to water and standard rodent chow.

Note that full information on the approval of the study protocol must also be provided in the manuscript.

ChIP-seq

Data deposition

☒ Confirm that both raw and final processed data have been deposited in a public database such as GEO.

☒ Confirm that you have deposited or provided access to graph files (e.g. BED files) for the called peaks.

Data access links *May remain private before publication.*	https://bigd.big.ac.cn/gsa/browse/CRA002490 and GEO accession GSE157339 https://www.ncbi.nlm.nih.gov/geo/query/acc.cgi?acc=GSE157339
Files in database submission	Fastq and bigwig files of ChIP-seq data
Genome browser session (e.g. UCSC)	Not available

Methodology

Replicates	2 biological replicates for MyoD, CTCF, H3K27ac ChIP-seq, no replicate for input data
Sequencing depth	30M reads for each MyoD and CTCF ChIP-Seq replicates and input. 20M reads for each H3K27ac ChIP-Seq replicates and input.
Antibodies	MyoD antibody: Santa Cruz, sc32758; H3K27ac antibody: Abcam, ab4729; CTCF antibody: Cell Signaling, 2899S
Peak calling parameters	Default parameters of MACS2
Data quality	Reads with a Phred quality score of <20 were removed. Non-unique reads were removed by SAMtools. Quality were accessed by deeptools and UCSC Genome Browser visualization and replicate clustering.
Software	Bowtie2 (v2.3.5), SAMtools (v1.9), MACS2 (v2.2.5), UCSC Genome Browser utility, DiffBind (v2.10.0), deeptools (v3.0.1), ChIPseeker (v1.20.0)

Flow Cytometry

Plots

Confirm that:

☒ The axis labels state the marker and fluorochrome used (e.g. CD4-FITC).

☒ The axis scales are clearly visible. Include numbers along axes only for bottom left plot of group (a 'group' is an analysis of identical markers).

☒ All plots are contour plots with outliers or pseudocolor plots.

☒ A numerical value for number of cells or percentage (with statistics) is provided.

Methodology

Sample preparation	Primary myoblasts were isolated from the hindlimb skeletal muscle of floxed MyoD mice at 2~3 weeks, minced and digested in a mixture of type II collagenase and Dispase B (Roche Applied Science). Cells were filtered from debris, centrifuged and cultured in growth medium (F-10 Ham's medium supplemented with 20% FBS, 10 ng/ml basic fibroblast growth factor, 1% penicillin– streptomycin and 1% glutamine) on collagen-coated cell culture plates at 37 °C in 5% CO2. The proliferating cells were infected with Cre- and GFP-expressing adenovirus (Ad-Cre). Solely GFP-expressing adenovirus served as control (Ad-ctrl). Then, the GFP positive cells were sorted 48 h post infection. The FACS-sorted GFP positive cells were plated at 60-70% confluence and switched to differentiation medium (DMEM with 2% horse serum) to induce differentiation for 24 h. Finally, the differentiating cells were collected and subjected to perform BL-HiC analysis.
Instrument	Flow cytometer ARIA III
Software	FlowJo 10.4, java version: 1.8.0_144-b01
Cell population abundance	76.1% of the cells were monocytes. Over 90% of the monocytes were GFP-positive cells.
Gating strategy	We selected SSC and FSC to determine the range of monocytes, as indicated in Supplementary Fig. 6D (left panel), then selected monocytes population using FSC/GFP-FITC to determine GFP positive cells, as indicated in Supplementary Fig. 6D (right panel). There was a clear boundary between GFP-positive and GFP-negative cell population.

☒ Tick this box to confirm that a figure exemplifying the gating strategy is provided in the Supplementary Information.
